# A Lecture to Teach an Approach and Improve Resident Comfort in Leading Resuscitation of Young Infants in the Emergency Department

**DOI:** 10.21980/J8H36J

**Published:** 2022-01-15

**Authors:** Anne Whitehead

**Affiliations:** * Indiana University School of Medicine, Department of Emergency Medicine, Division of Pediatric Emergency Medicine, Indianapolis, IN

## Abstract

**Audience:**

The intended audience of this lecture is emergency medicine residents at all levels of training. It is also appropriate for practicing emergency physicians interested in improving comfort in resuscitating sick young infants, ages 0–60 days.

**Introduction:**

The majority of sick and injured children in the United States are seen and treated in general emergency departments.^1^ This includes very young infants (0–60 days old) in need of immediate resuscitation. Resuscitation of children in this age group involves use of specific knowledge and skills that residents and emergency physicians in general have fewer opportunities to practice.^2^,^3^ Emergency medicine residents and practicing emergency physicians often report this as an area of particular discomfort in practice.^4^,^5^ It is important that the inconsistent and infrequent opportunities to resuscitate young infants during emergency medicine residency and beyond are supplemented by residency didactics that focus on improving comfort and skills with this population of sick children. This lecture focuses on a practical approach intended to improve the relevant knowledge, skills, and confidence required to stabilize a critically ill young infant in a general emergency department.

**Educational Objectives:**

By the end of this lecture, participants should be able to:

1. Apply a consistent approach to the initial resuscitation of a critically ill young infant in the emergency department.
2. Select appropriate medications and equipment for use in resuscitation of critically ill young infants.
3. Describe the components of the Pediatric Assessment Triangle,^6^ which can be used to identify critically ill infants and children.
4. Improve comfort in resuscitating young infants in the emergency department.

**Educational Methods:**

This is a live lecture format using PowerPoint slides. The lecture emphasizes a practical approach to improve the skills and knowledge required for successful young infant resuscitation. It utilizes a case-based approach, and encourages the audience to determine next steps in care to mimic the real time decision-making required for care of critically ill young infants in the ED.

**Research Methods:**

Learners were asked to fill out anonymous pre- and post quizzes immediately prior to and directly after the lecture was given. These surveys included questions to assess resident knowledge as well as resident comfort as it pertained to resuscitation of critically ill young infants.

**Results:**

Resident comfort with resuscitation of young infants improved with a mean Standard Deviation (SD) pre-lecture rating of 23.1(14.9) on a 100-point visual analog scale and a mean (SD) post lecture rating of 46.7(14.6). Resident performance on all knowledge base questions improved on the post-lecture quiz for all four questions asked.

**Discussion:**

This lecture was effective in improving emergency medicine resident comfort and practical knowledge pertaining to resuscitation of young infants in the emergency department. The emphasis on a practical approach was well received by the resident audience, and they engaged well with audience participation portions of the lecture. The impact of the lecture can be enhanced by having the lecturer share their own real-world experience of resuscitation of young infants in the emergency department during the discussion portions of the lecture.

**Topics:**

Neonatal resuscitation, infant resuscitation, pediatric assessment triangle, neonatal sepsis, congenital heart disease, congenital adrenal hyperplasia, non-accidental trauma, malrotation.

## USER GUIDE

[Table t1-jetem-7-1-l11]

**Table t1-jetem-7-1-l11:** 

List of Resources:	
Abstract	11
User Guide	13
Crashing Neonates Lecture	15
Pre-Test Questions	16
Post-Test Questions	17
Test Answers	18

**Learner Audience:**

Interns, Junior Residents, Senior Residents

**Time Required for Implementation:** 30 minutes

**Recommended Number of Learners per Instructor:**

20–60 learners

**Topics:**

Neonatal resuscitation, infant resuscitation, pediatric assessment triangle, neonatal sepsis, congenital heart disease, congenital adrenal hyperplasia, non-accidental trauma, malrotation.

**Objectives:**

By the end of this lecture, participants should be able to:

1. Describe the components of the Pediatric Assessment Triangle,^6^ which can be used to identify critically ill infants and children.
2. Select appropriate medications and equipment for use in resuscitation of critically ill young infants.
3. Apply a consistent approach to the initial resuscitation of a critically ill young infant in the emergency department.
4. Improve learner comfort in resuscitating young infants in the emergency department.

### Linked objectives and methods

The lecture emphasizes a practical approach to improve the skills and knowledge required for successful young infant resuscitation. It utilizes a case-based approach, and encourages the audience to determine next steps in care to mimic the real time decision-making required for care of critically ill young infants in the ED. Learners are encouraged to take an active role during the frequent planned pauses for audience participation, to allow them to mentally rehearse the approach to resuscitation with the resources they are likely to have available in clinical practice. The lecture avoids in-depth discussion of pathophysiology, and de-emphasizes the importance of memorization of facts, to better achieve the more practical, skills-based objectives.

### Recommended pre-reading for instructor

- Morgenstern J. Resuscitation of the crashing infant (pediatric resuscitation). https://first10em.com/crashing-infant/7

### Results

A faculty member trained and practicing in emergency medicine and pediatric emergency medicine delivered this lecture to emergency medicine residents during a regularly scheduled resident didactic conference during a 30-minute lecture slot. Thirty residents, representing all levels of training, were in attendance in person, with additional residents viewing remotely via a secure Zoom. Five minutes prior to the start of the lecture, residents were provided a QR code which linked to a 5 question, anonymous pre-lecture quiz. They were provided with a separate QR code after the lecture linking to the post-lecture quiz, which consisted of the same questions as the pre-lecture quiz. 23 residents completed the pre-lecture quiz, and 24 completed the post lecture quiz.

Both comfort and performance on knowledge questions improved after the lecture. Residents reported low comfort with young infant resuscitation on the pre-lecture quiz, with a mean (SD) of 23.1 (14.9), and increased to a mean (SD) of 46.7(14.6) after the lecture. There were 4 knowledge related questions on the quiz, and while 2 had a relatively high percentage of residents answering correctly even on the pre-lecture quiz, the percent of residents answering correctly improved for all 4 questions on the post-lecture quiz (Figure 1)

**Figure:**
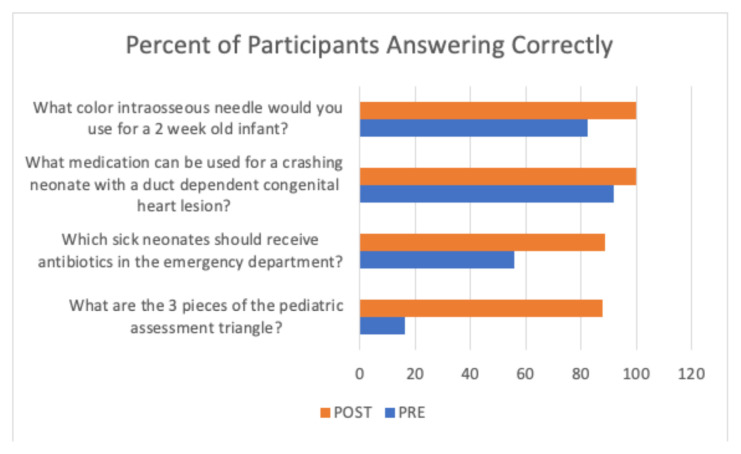


Residents commented that they found the lecture to be valuable. One resident reported to the lecturer that the skills gained had been helpful in caring for a sick young infant they had the opportunity to care for during an emergency department shift the day after the lecture occurred.

### Tips for Successful Implementation

This lecture allowed for excellent audience engagement and participation. The audience was frequently asked open ended questions during lecture, and in-person participants consistently answered questions and shared their prior experiences with infant resuscitation. There was some, but not as robust, participation from the online audience. Were this lecture format to be used in a virtual only format, it might be important to use techniques to improve audience participation, such as an online audience response system, a polling feature, and/or a chat moderator.

This lecture is best delivered by a lecturer with experience and expertise in infant resuscitation. Audience members often asked questions that called upon the lecturer’s real-world experience and clinical judgement. Lecturers might consider replacing some of the details of the case described, and relevant images used in this lecture, with those of cases they themselves have seen in their own clinical practice.

### Associated content (optional)

- Lecture PowerPoint
- Lecture Pre-Test Questions
- Lecture Post-Test Questions
- Lecture Pre/Post-Test Answers

### Technology necessary

This lecture requires a computer and projector for delivery of the lecture slides.

## Supplementary Information



## Figures and Tables

**Figure f2-jetem-7-1-l11:**
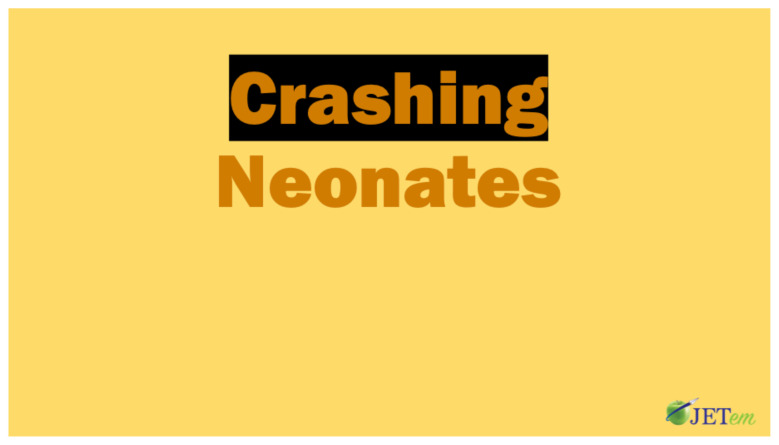
Please see associated PowerPoint file

**Figure f3-jetem-7-1-l11:**
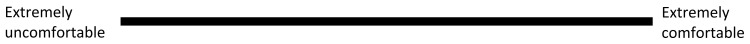


**Figure f4-jetem-7-1-l11:**
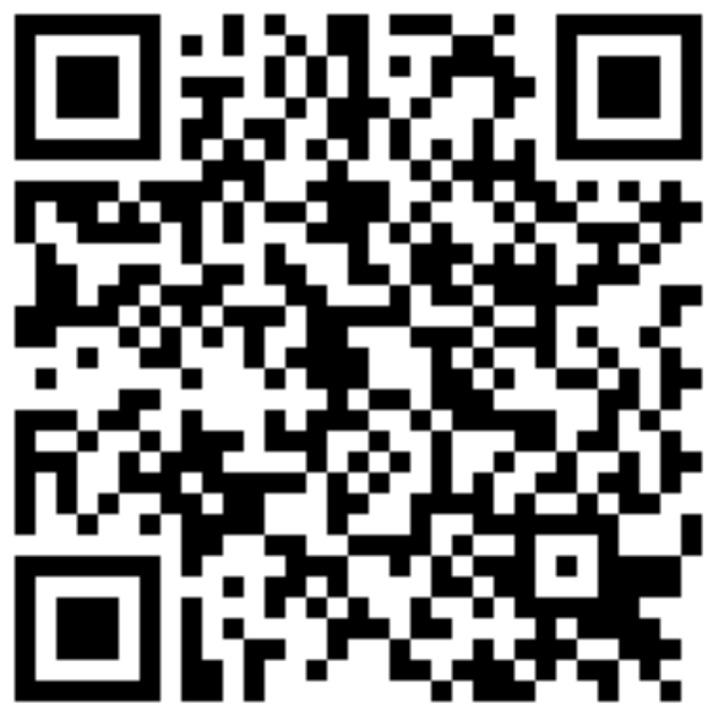


**Figure f5-jetem-7-1-l11:**
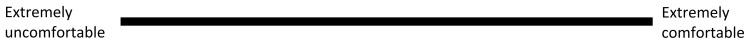


## LEARNER MATERIALS

### Crashing Neonates Lecture

[Fig f2-jetem-7-1-l11]

### Pre-Test Questions

What are the 3 sides of the pediatric assessment triangle?

Which sick neonates/young infants should receive antibiotics in the emergency department?

What medication can be used for a crashing neonate with a duct dependent congenital heart lesion?

What color intraosseous needle would you use for a 2-week-old infant?

How comfortable do you feel taking care of sick neonates/young infants in the emergency department? Place a mark on the line:[Fig f3-jetem-7-1-l11]

### Post-Test Questions

Please fill out the anonymous “post” quiz to the best of your ability **after** lecture. You can fill out this paper, or use the QR code:[Fig f4-jetem-7-1-l11]

What are the 3 sides of the pediatric assessment triangle?

Which sick neonates/young infants should receive antibiotics in the emergency department?

What medication can be used for a crashing neonate with a duct dependent congenital heart lesion?

What color intraosseous needle would you use for a 2-week-old infant?

How comfortable do you feel taking care of sick neonates/young infants in the emergency department? Place a mark on the line:[Fig f5-jetem-7-1-l11]

### Pre/Post-Test Answers

What are the 3 sides of the pediatric assessment triangle?

***A: Appearance, work of breathing, perfusion to the skin***

Which sick neonates/young infants should receive antibiotics in the emergency department?

***A: All sick/unstable neonates should receive antibiotics empirically***

What medication can be used for a crashing neonate with a duct dependent congenital heart lesion?

***A: Prostaglandin (or PGE or alprostadil)***

What color intraosseous needle would you use for a 2-week-old infant?

***A: Pink***
