# Supplementary material for: A Lecture to Teach an Approach and Improve Resident Comfort in Leading Resuscitation of Young Infants in the Emergency Department
Source: J Educ Teach Emerg Med. 2022 Jan 15;7(1):L11–8. doi: 10.21980/J8H36J (PMC10358869; doi:10.21980/J8H36J)
Supplement: Supplementary file 1 [file JETem-7-1-L11-supp1.pptx]

## Slide 1
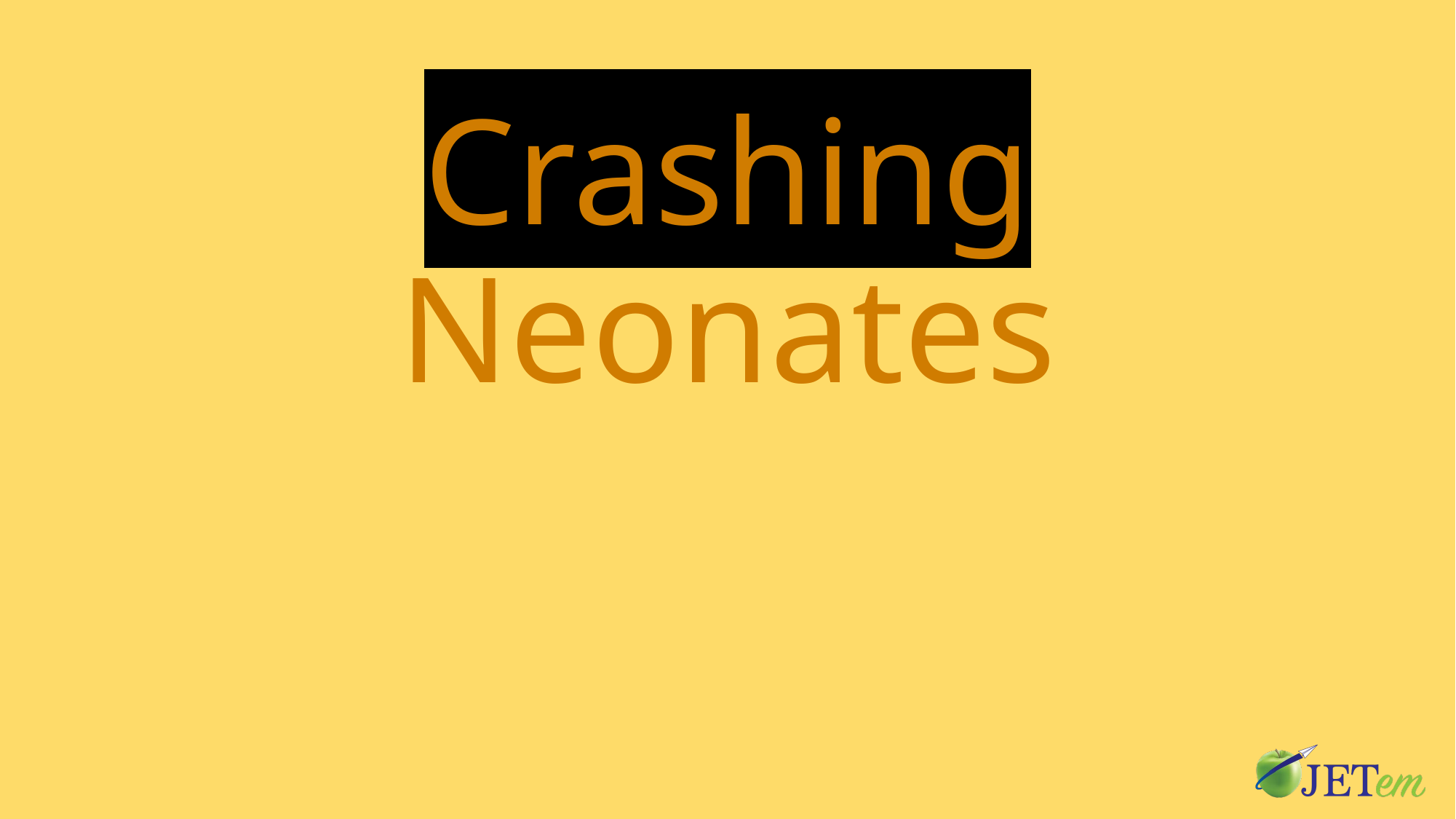

# Crashing Neonates

## Slide 2
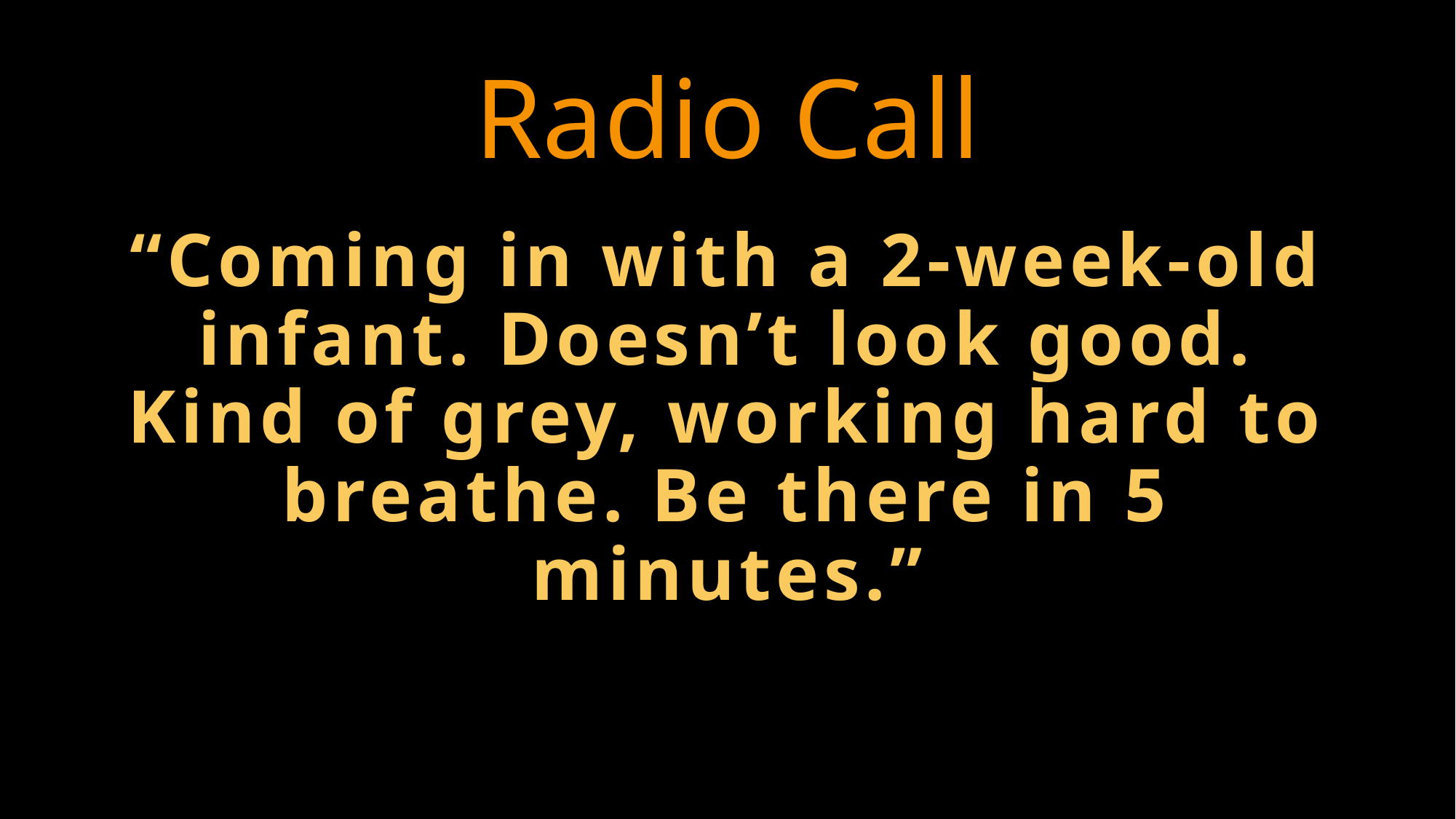

# Radio Call
“Coming in with a 2-week-old infant. Doesn’t look good. Kind of grey, working hard to breathe. Be there in 5 minutes.”

## Slide 3
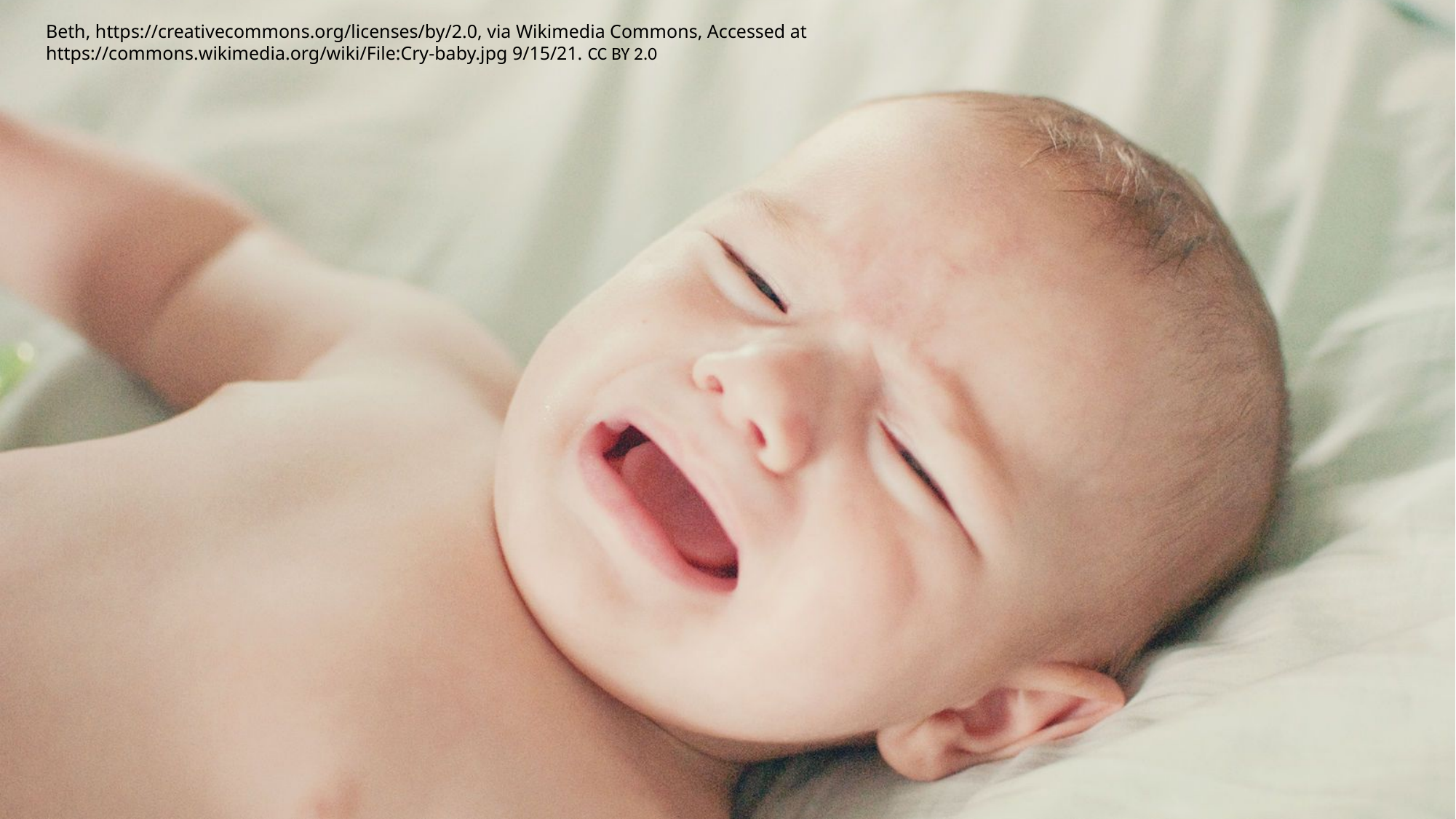

Beth, https://creativecommons.org/licenses/by/2.0, via Wikimedia Commons, Accessed at https://commons.wikimedia.org/wiki/File:Cry-baby.jpg 9/15/21. CC BY 2.0

## Slide 4
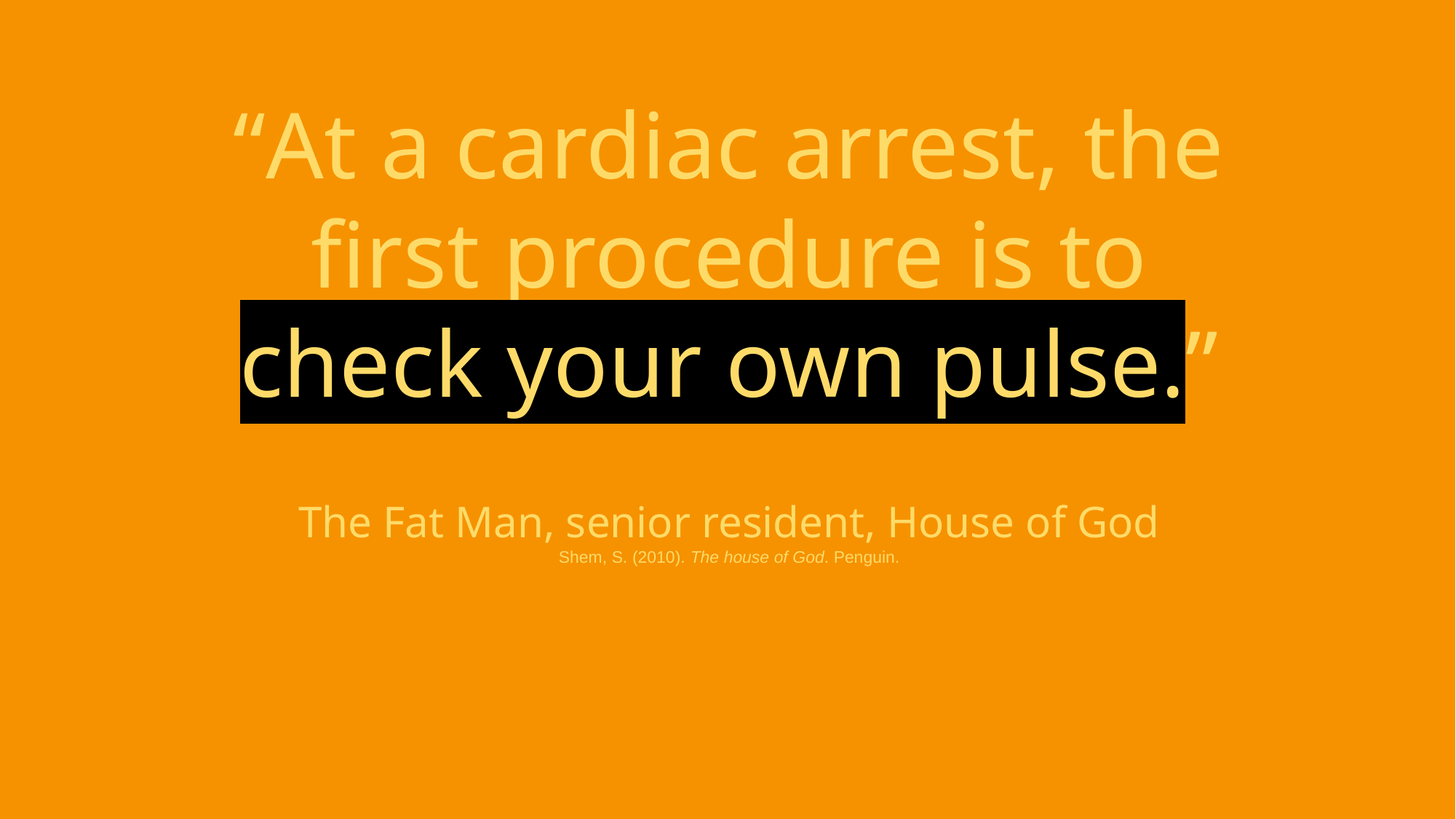

“At a cardiac arrest, the first procedure is to check your own pulse.”
The Fat Man, senior resident, House of God
Shem, S. (2010). The house of God. Penguin.

## Slide 5
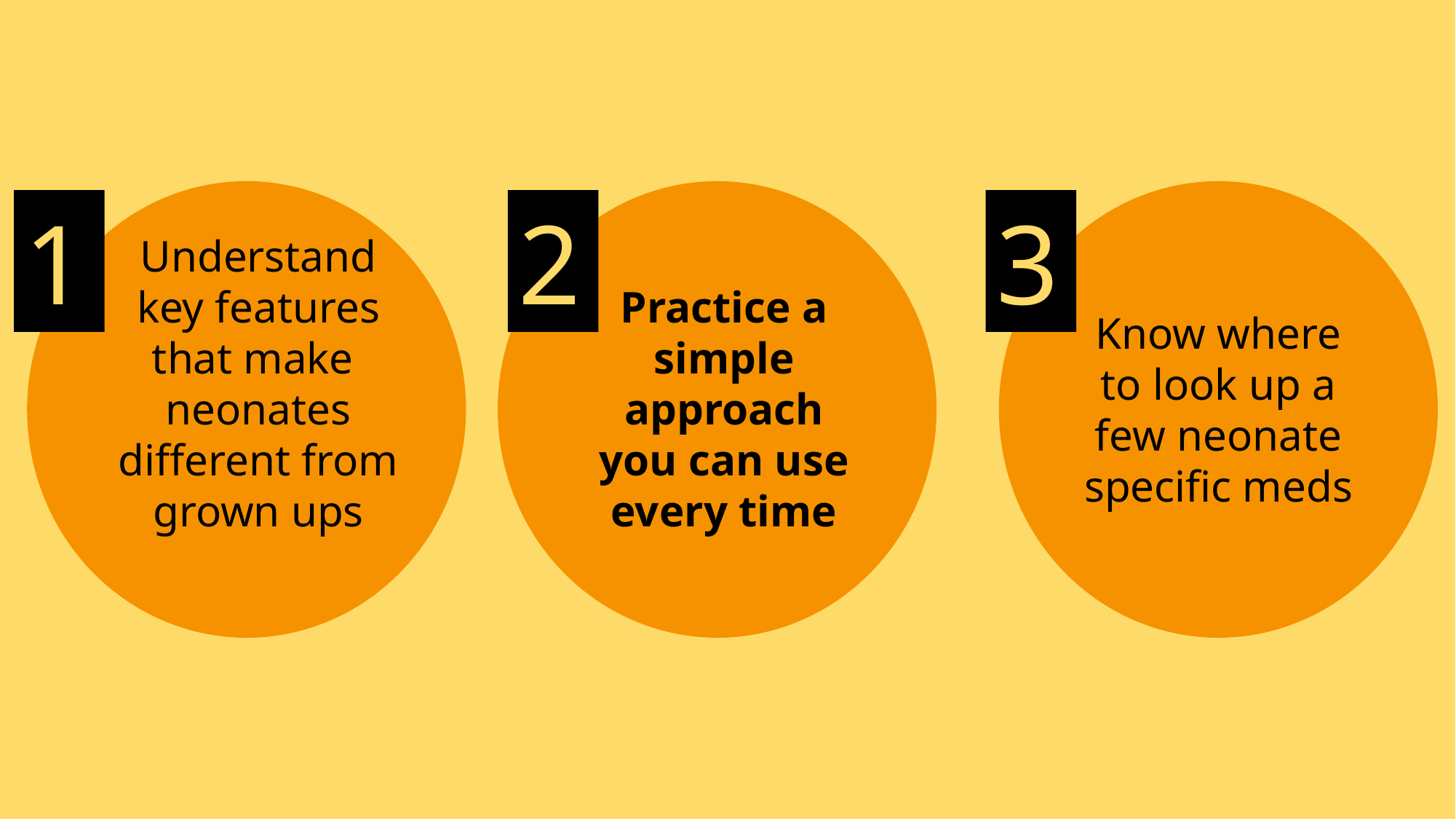

1
3
2
Understand key features that make neonates different from grown ups
Practice a simple approach you can use every time
Know where to look up a few neonate specific meds

## Slide 6
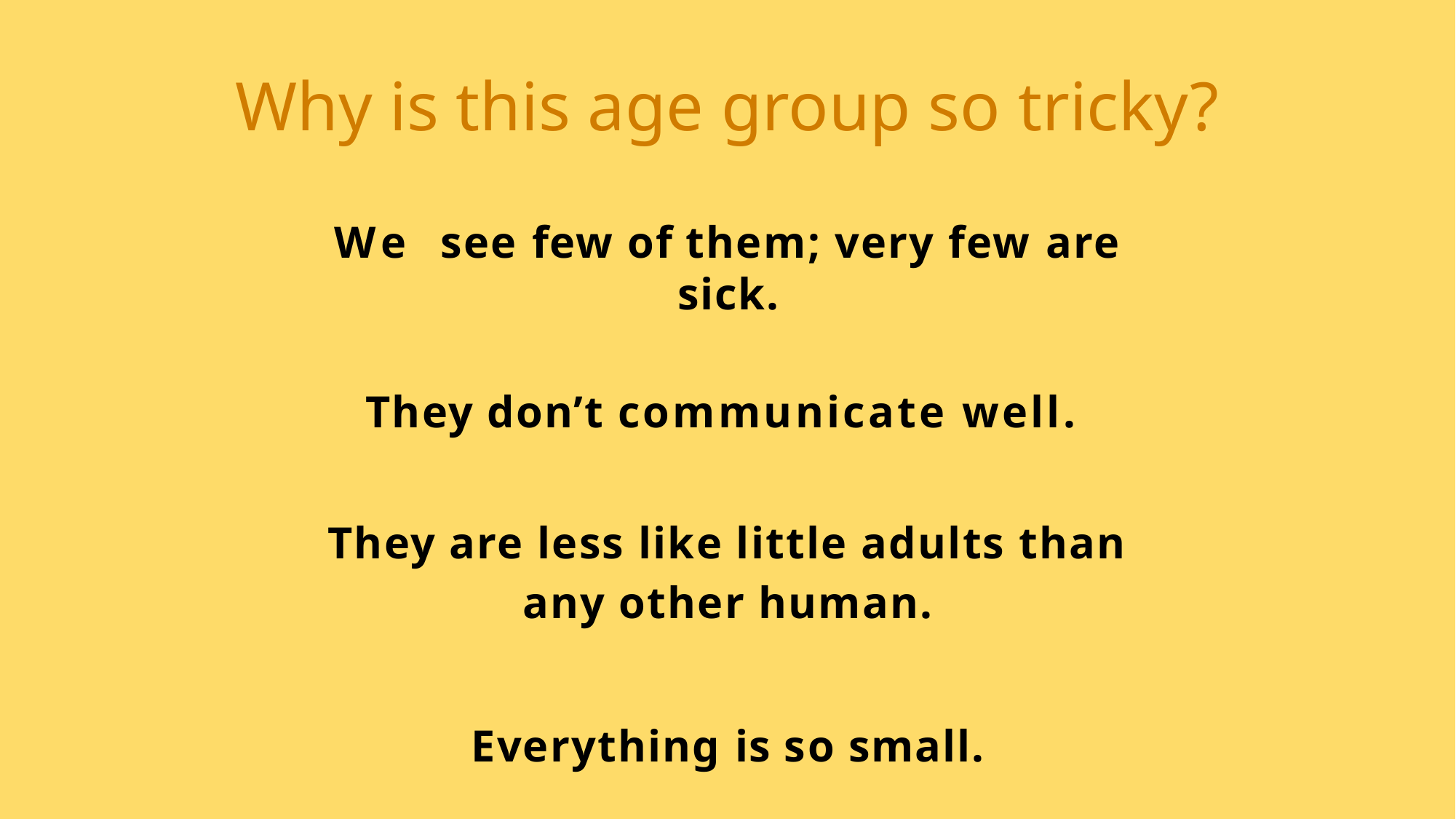

# Why is this age group so tricky?
We see few of them; very few are sick.
They don’t communicate well.
They are less like little adults than any other human.
Everything is so small.

## Slide 7
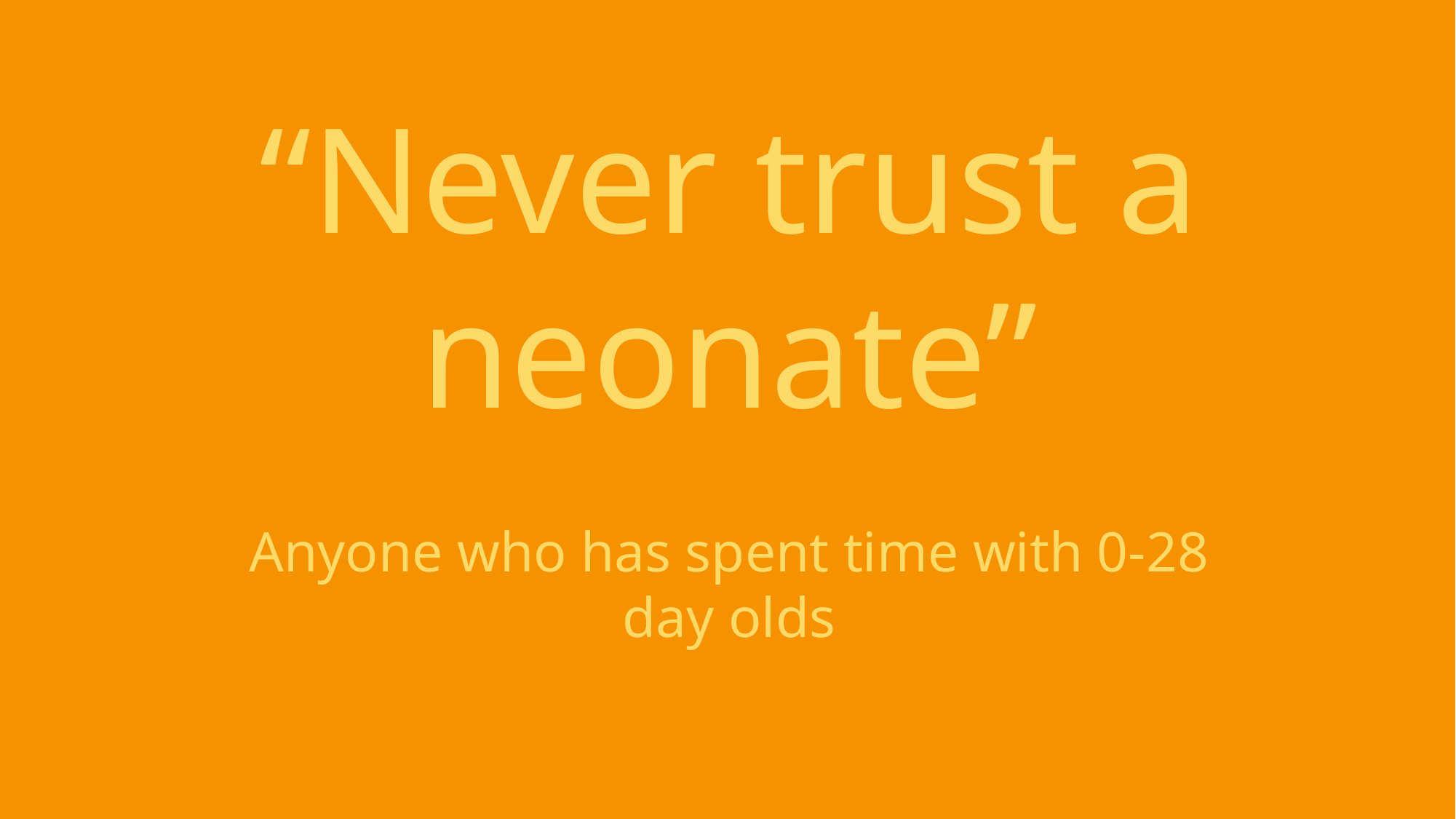

“Never trust a neonate”
Anyone who has spent time with 0-28 day olds

## Slide 8
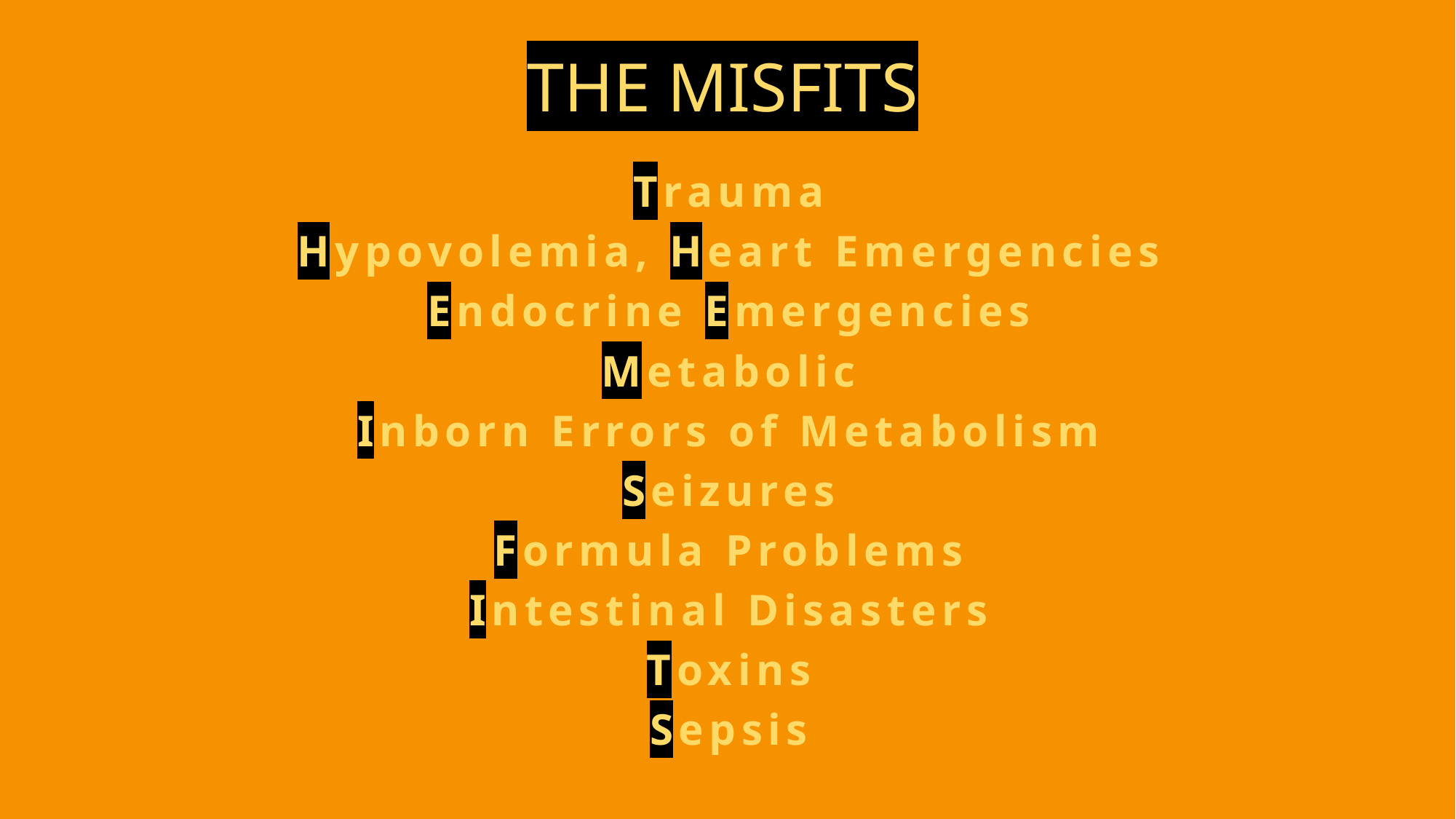

# THE MISFITS
Trauma
Hypovolemia, Heart Emergencies
Endocrine Emergencies
Metabolic
Inborn Errors of Metabolism
Seizures
Formula Problems
Intestinal Disasters
Toxins
Sepsis

## Slide 9
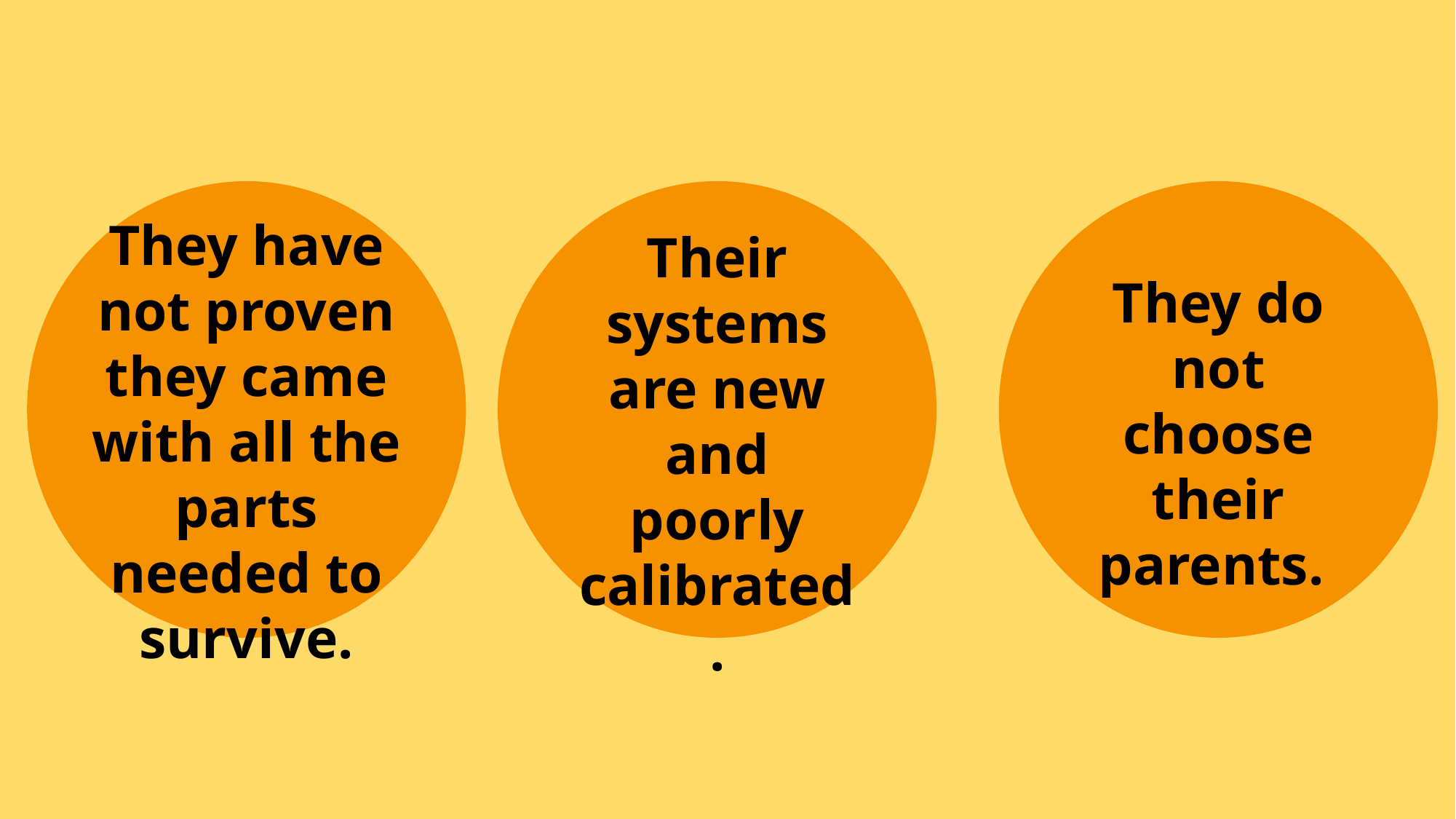

They have not proven they came with all the parts needed to survive.
Their systems are new and poorly calibrated.
They do not choose their parents.

## Slide 10
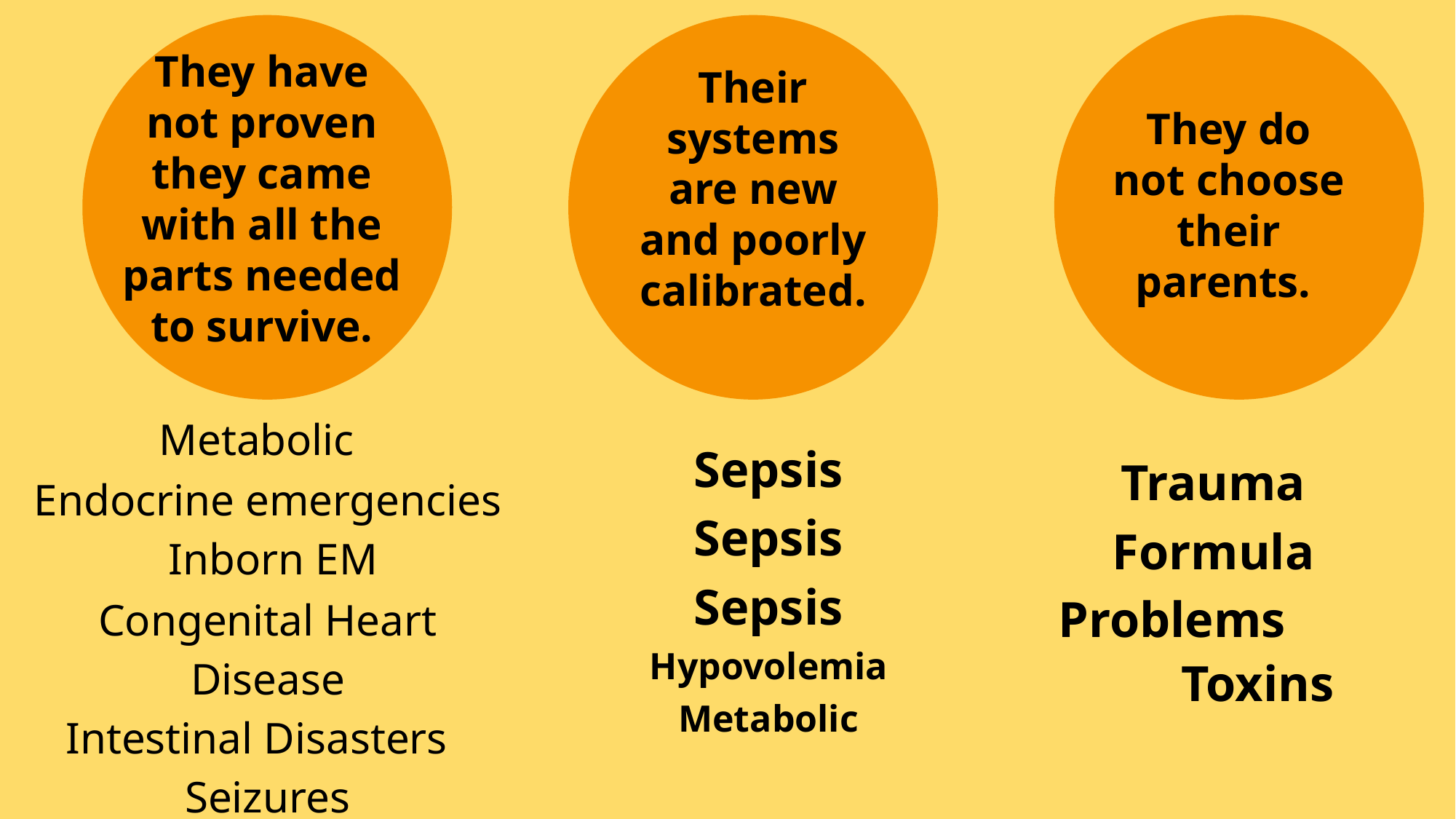

They have not proven they came with all the parts needed to survive.
Their systems are new and poorly calibrated.
They do not choose their parents.
Metabolic
Endocrine emergencies Inborn EM
Congenital Heart Disease
Intestinal Disasters
Seizures
Sepsis
Sepsis
Sepsis
Hypovolemia
Metabolic
Trauma
Formula Problems
Toxins

## Slide 11
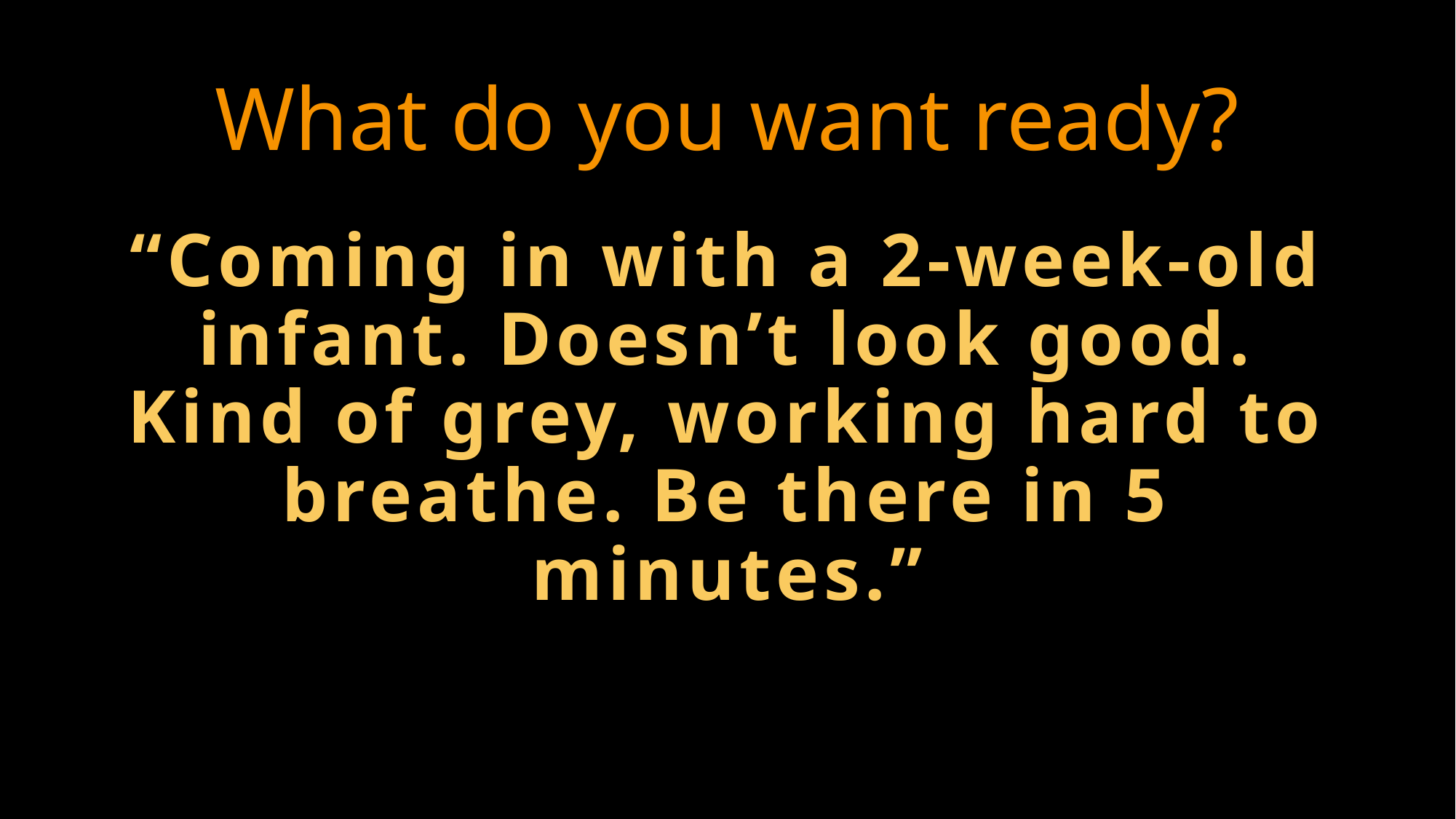

# What do you want ready?
“Coming in with a 2-week-old infant. Doesn’t look good. Kind of grey, working hard to breathe. Be there in 5 minutes.”

## Slide 12
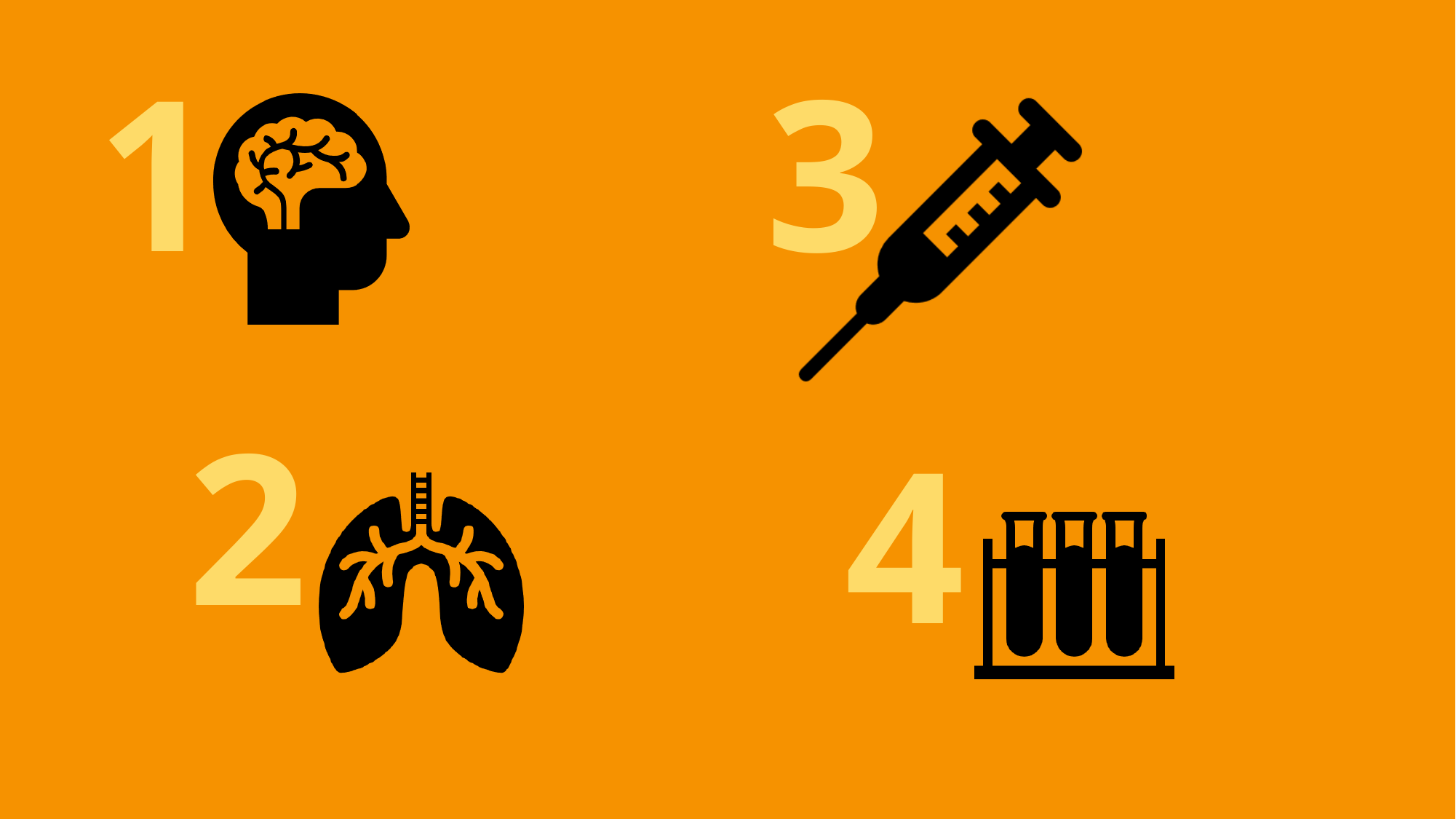

1
3
2
4

## Slide 13
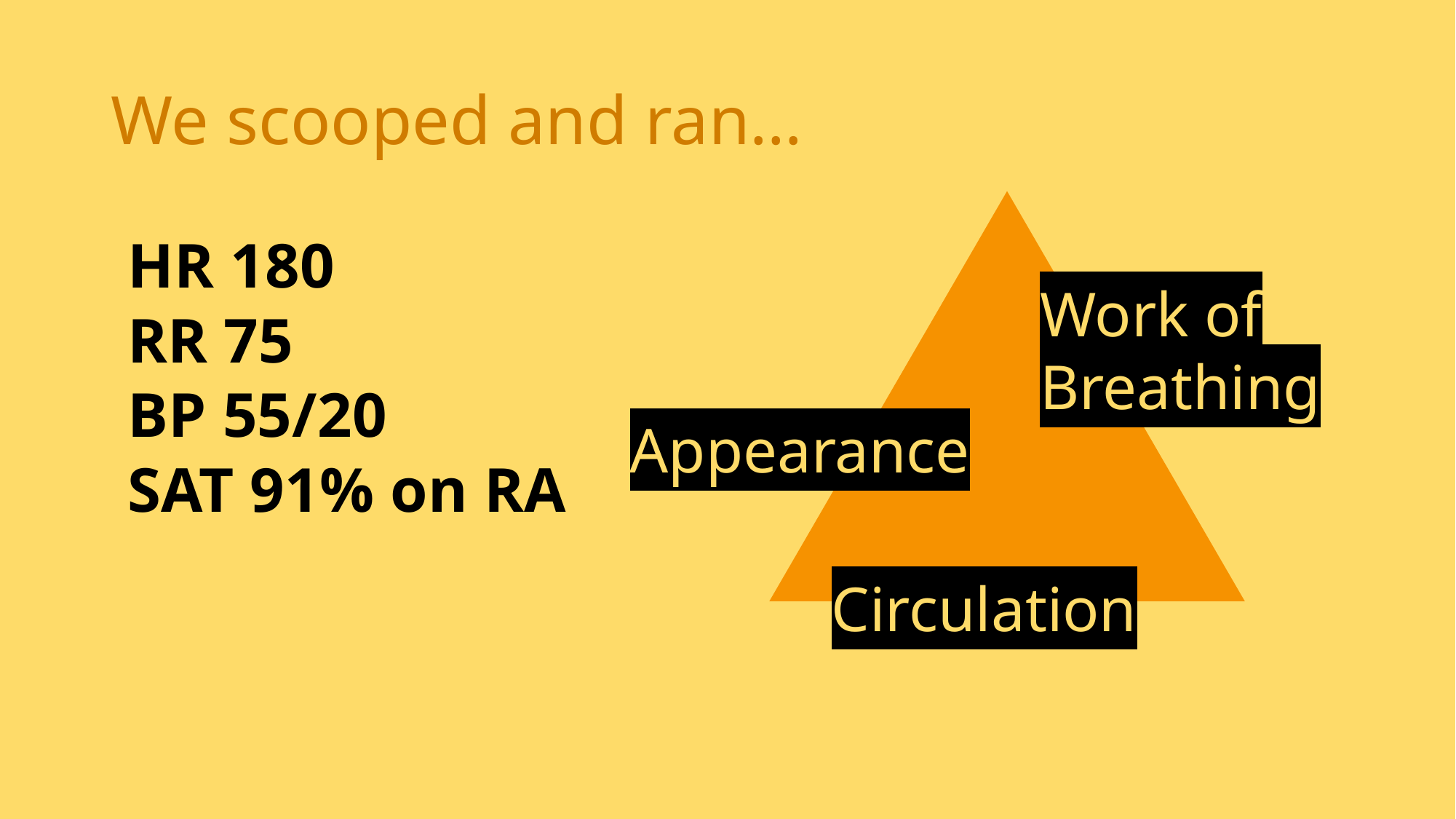

# We scooped and ran…
HR 180
RR 75
BP 55/20
SAT 91% on RA
Work of Breathing
Appearance
Circulation

## Slide 14
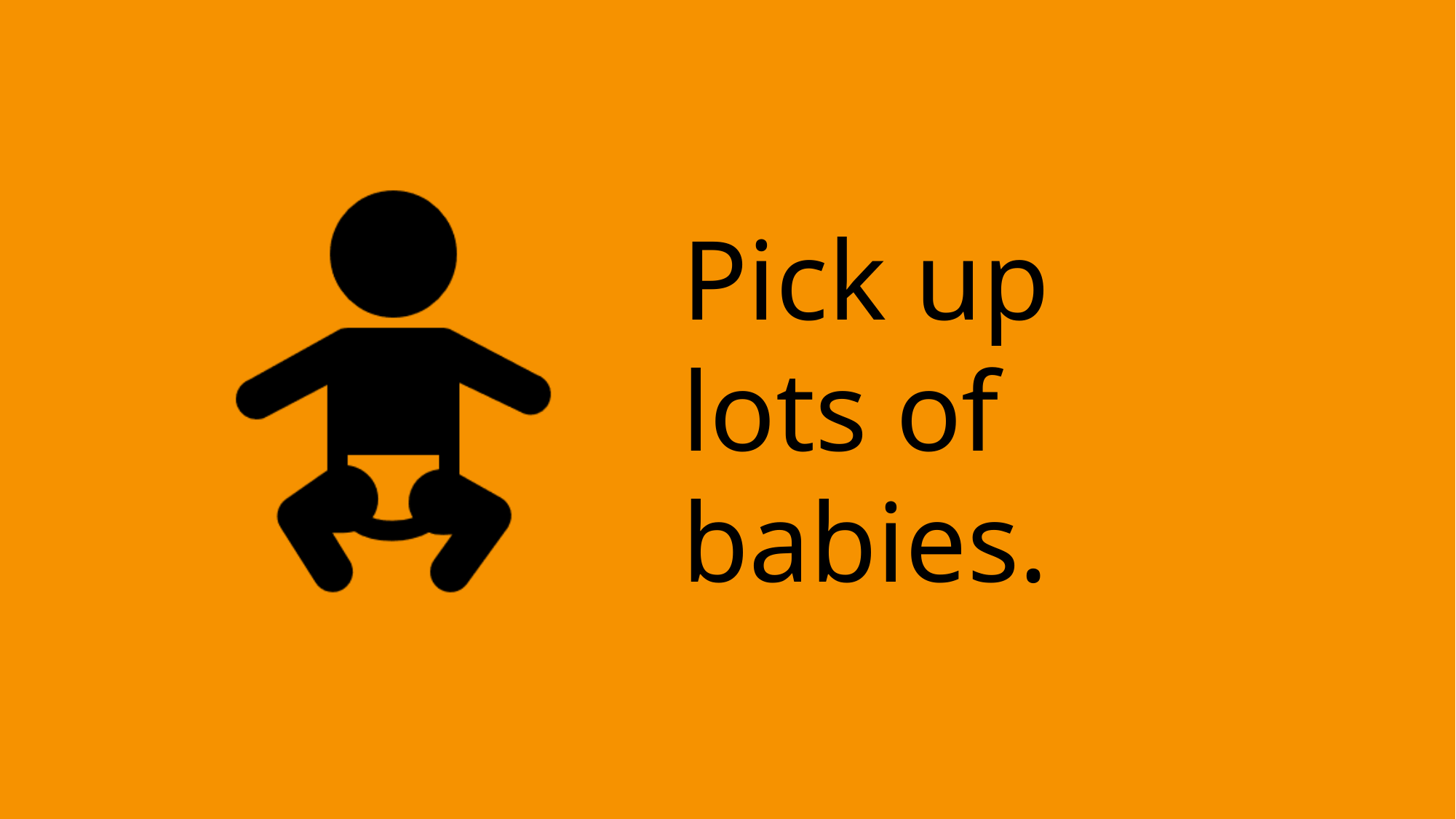

Pick up lots of babies.

## Slide 15
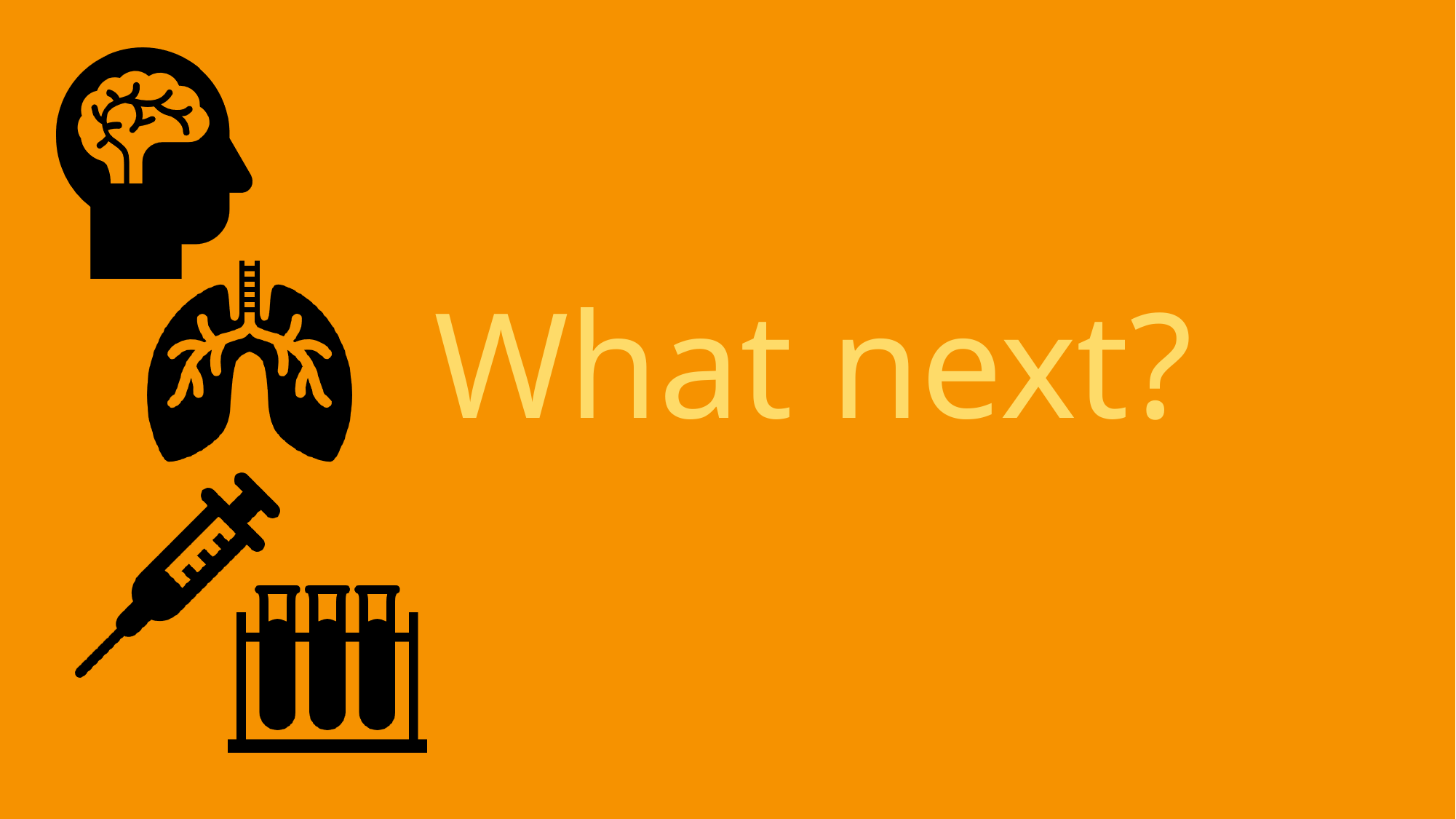

What next?

## Slide 16
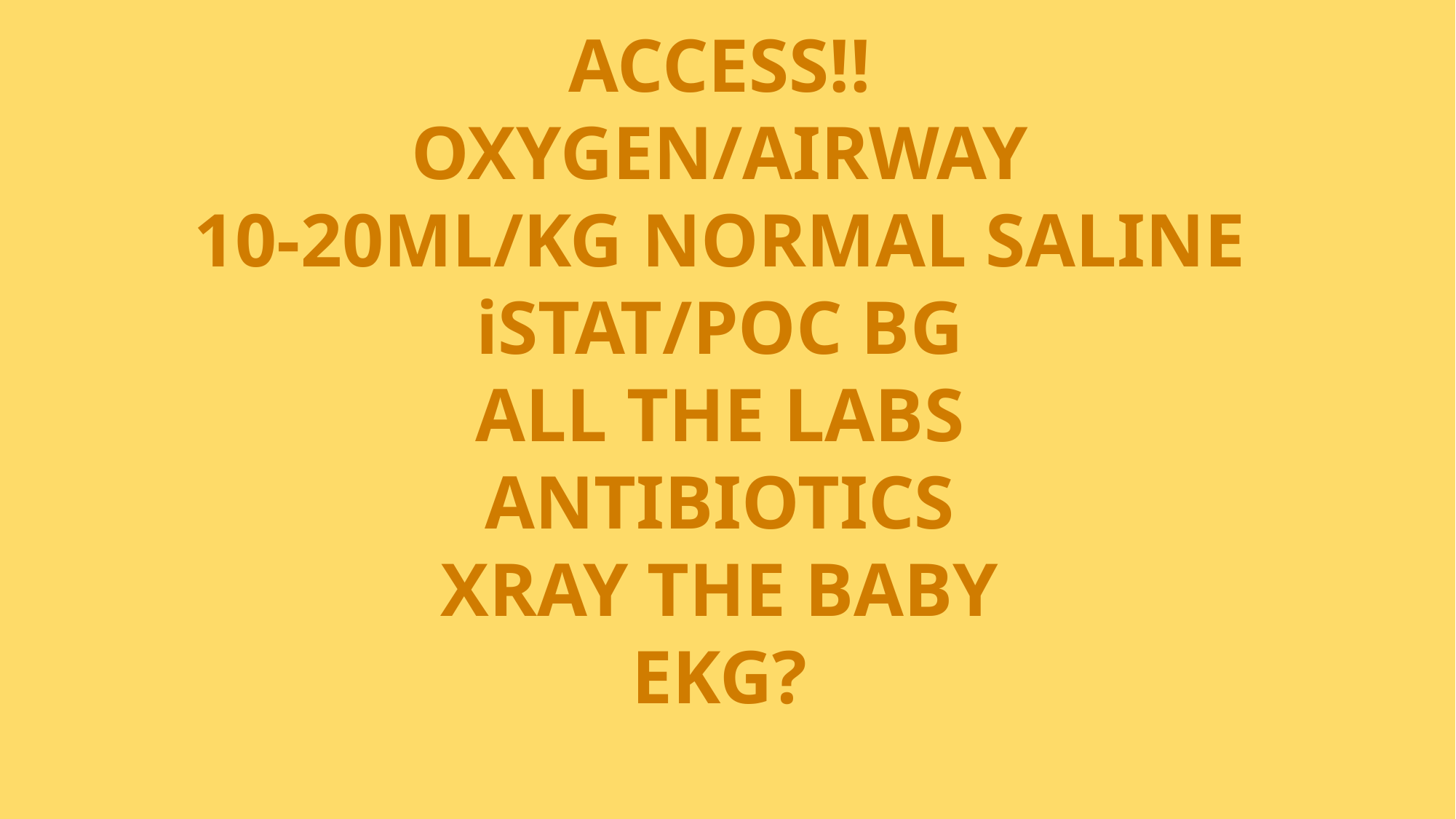

ACCESS!!
OXYGEN/AIRWAY
10-20ML/KG NORMAL SALINE
iSTAT/POC BG
ALL THE LABS
ANTIBIOTICS
XRAY THE BABY
EKG?

## Slide 17
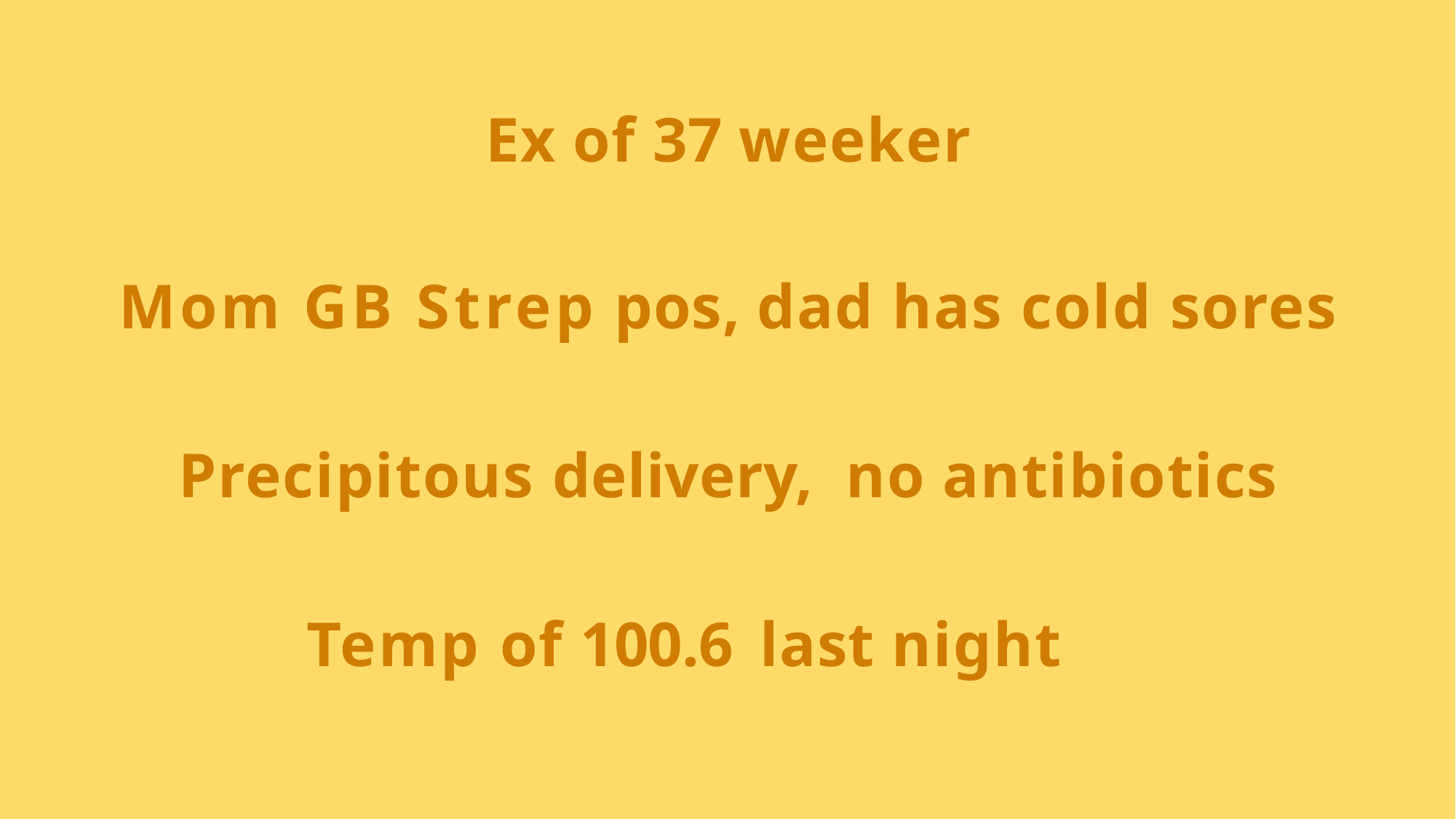

Ex of 37 weeker
Mom GB Strep pos, dad has cold sores
Precipitous delivery, no antibiotics
Temp of 100.6 last night

## Slide 18
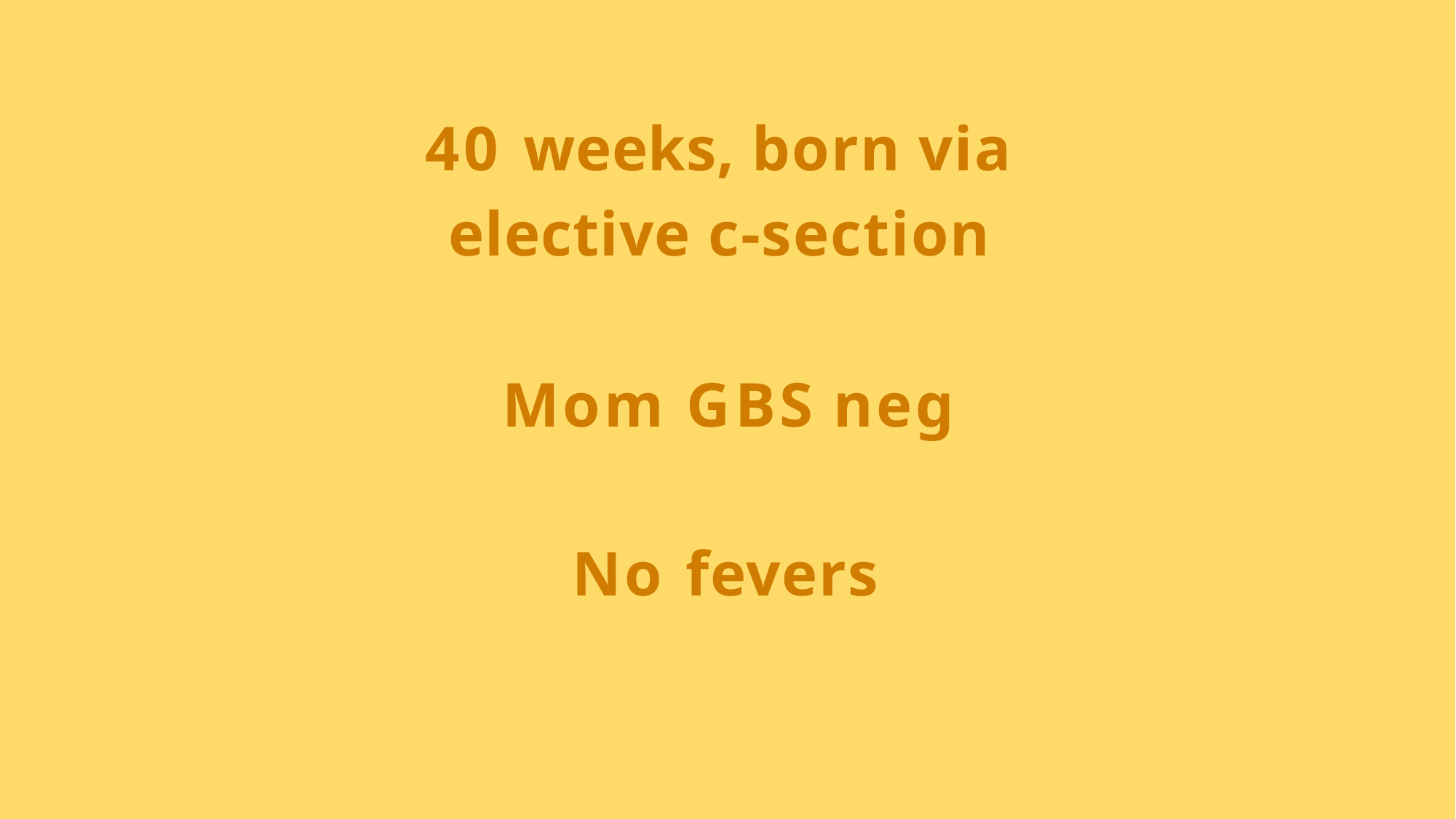

40 weeks, born via elective c-section
Mom GBS neg
No fevers

## Slide 19
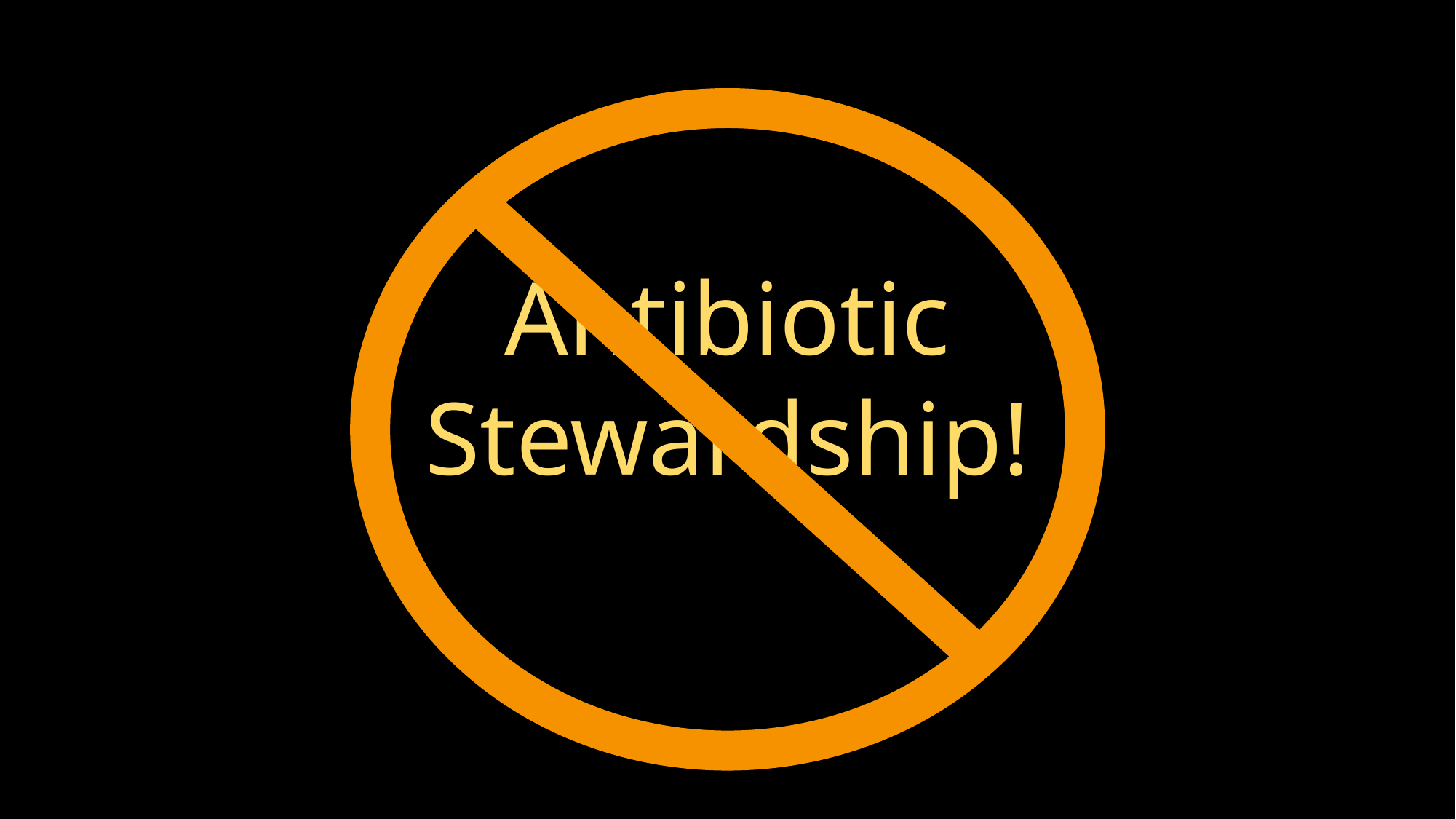

Antibiotic Stewardship!

## Slide 20
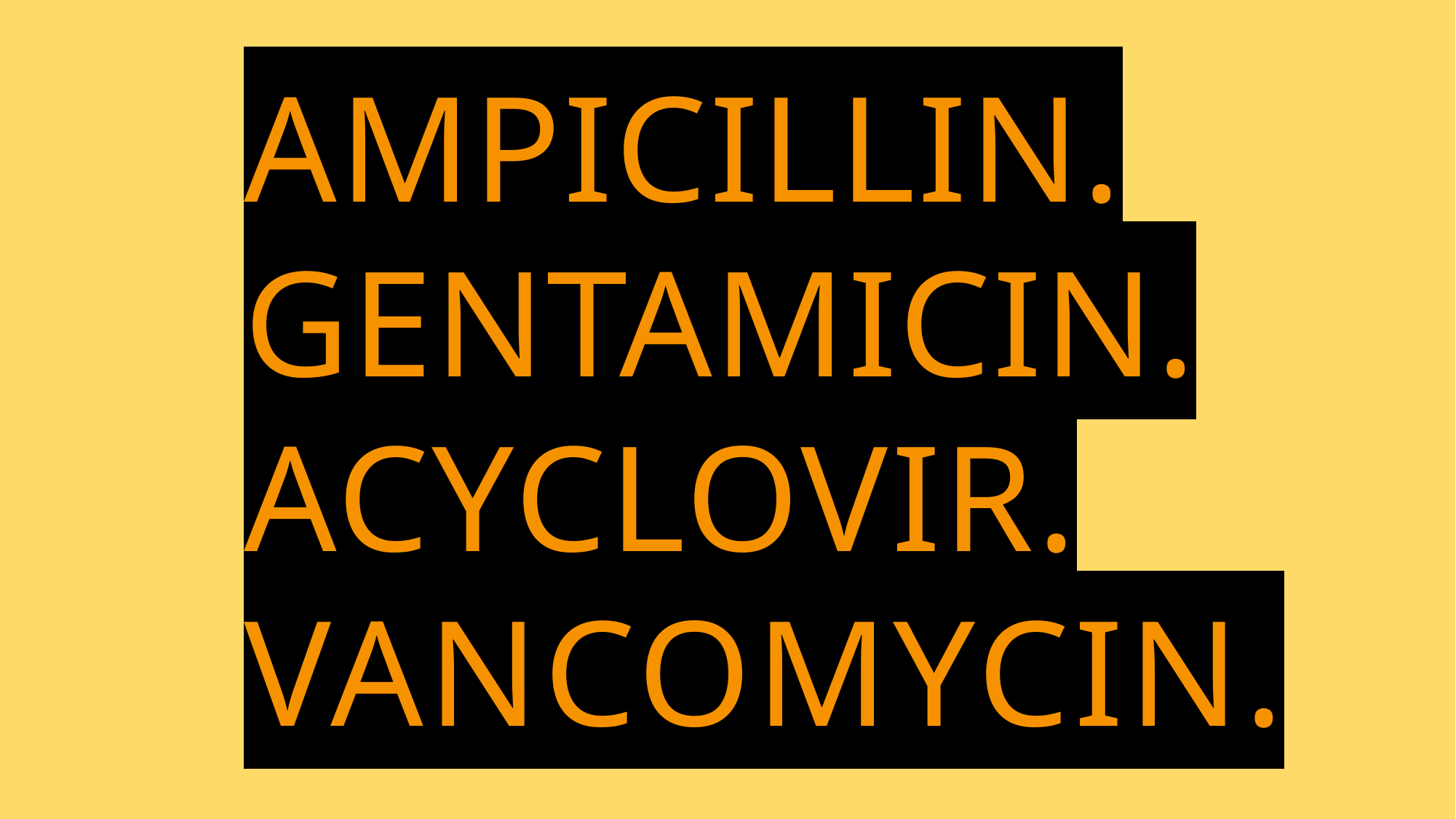

AMPICILLIN. GENTAMICIN. ACYCLOVIR. VANCOMYCIN.

## Slide 21
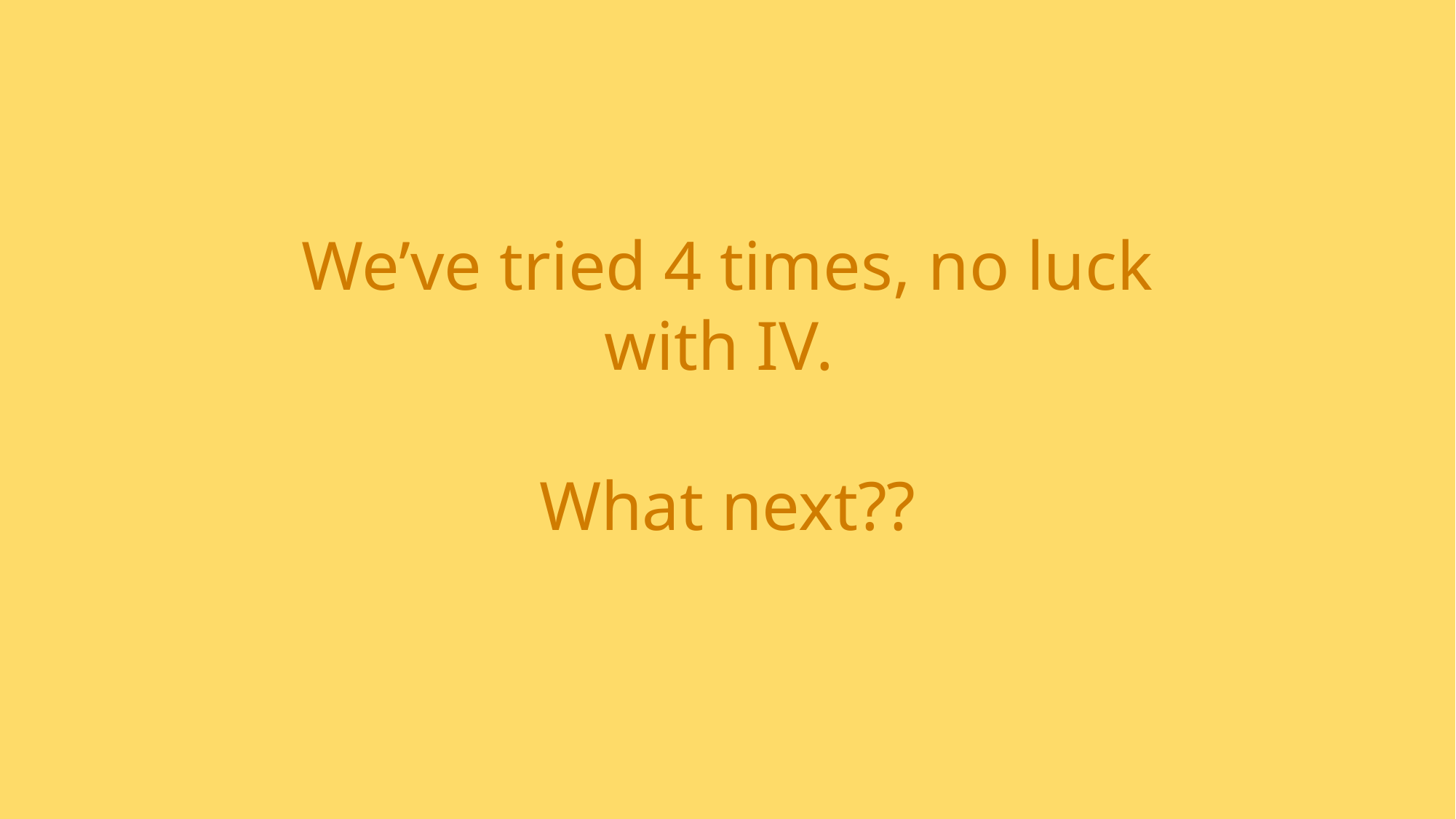

We’ve tried 4 times, no luck with IV.
What next??

## Slide 22
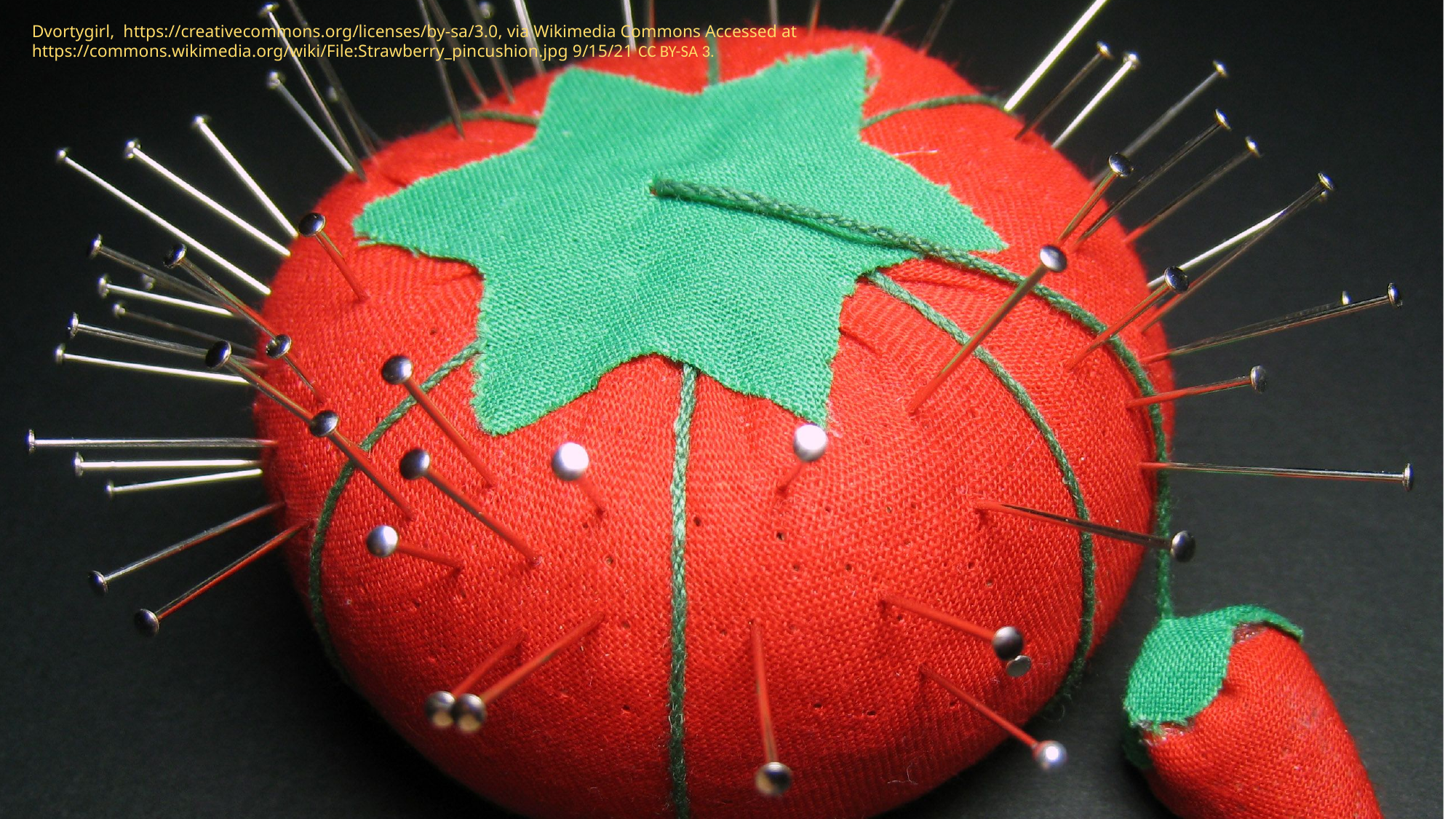

Dvortygirl,  https://creativecommons.org/licenses/by-sa/3.0, via Wikimedia Commons Accessed at https://commons.wikimedia.org/wiki/File:Strawberry_pincushion.jpg 9/15/21 CC BY-SA 3.

## Slide 23
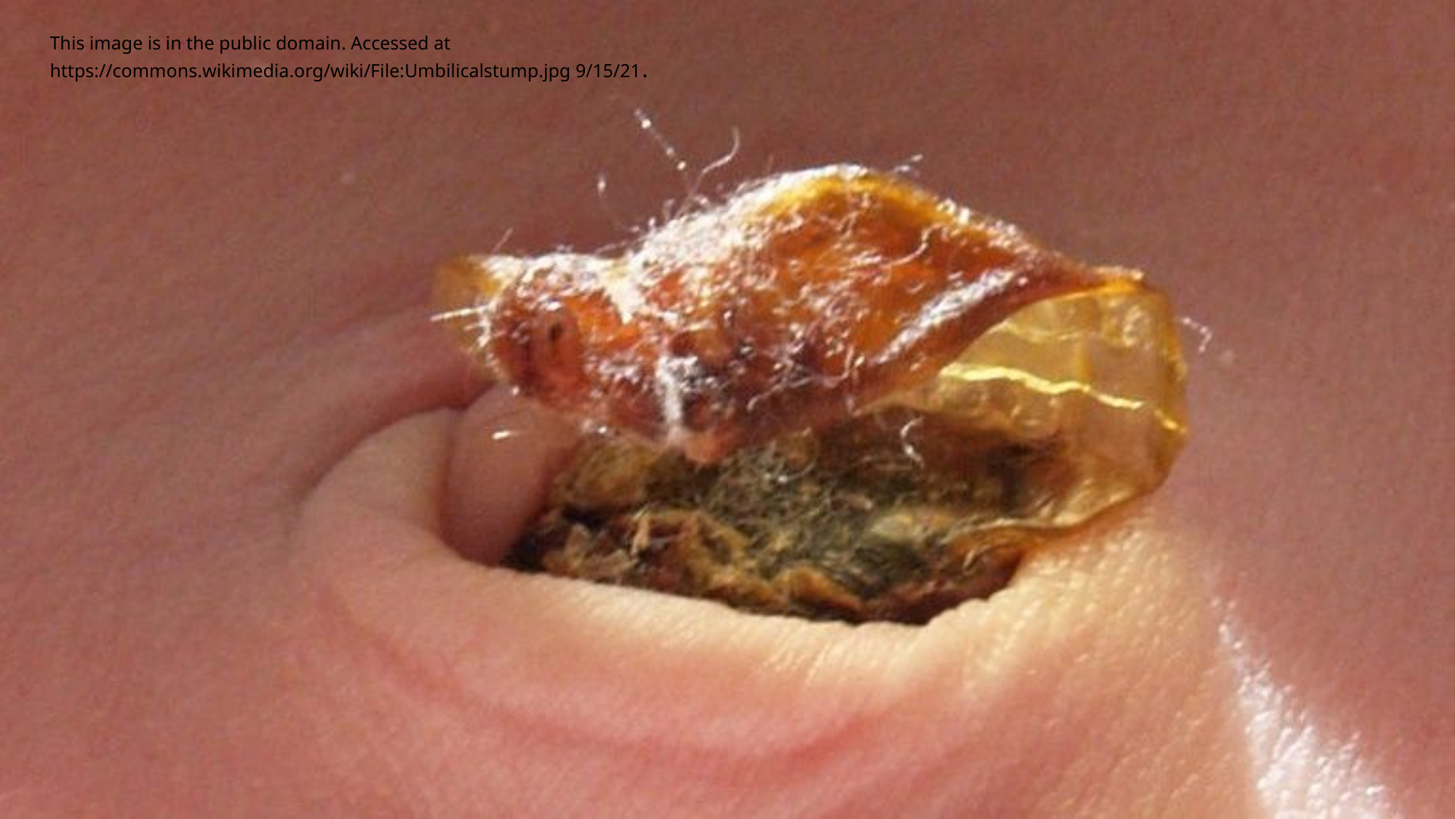

This image is in the public domain. Accessed at https://commons.wikimedia.org/wiki/File:Umbilicalstump.jpg 9/15/21.

## Slide 24
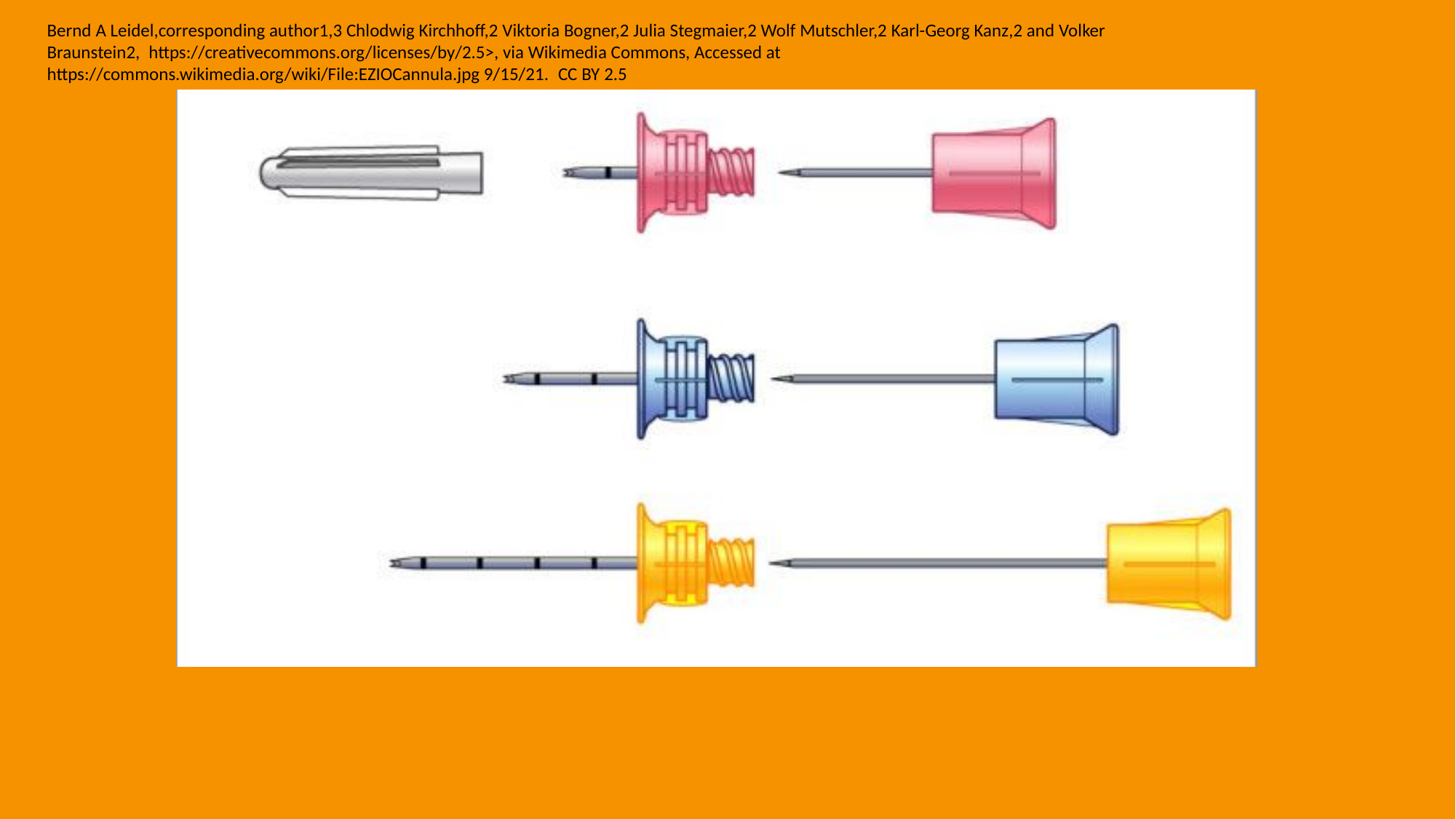

Bernd A Leidel,corresponding author1,3 Chlodwig Kirchhoff,2 Viktoria Bogner,2 Julia Stegmaier,2 Wolf Mutschler,2 Karl-Georg Kanz,2 and Volker Braunstein2,  https://creativecommons.org/licenses/by/2.5>, via Wikimedia Commons, Accessed at https://commons.wikimedia.org/wiki/File:EZIOCannula.jpg 9/15/21.  CC BY 2.5

## Slide 25
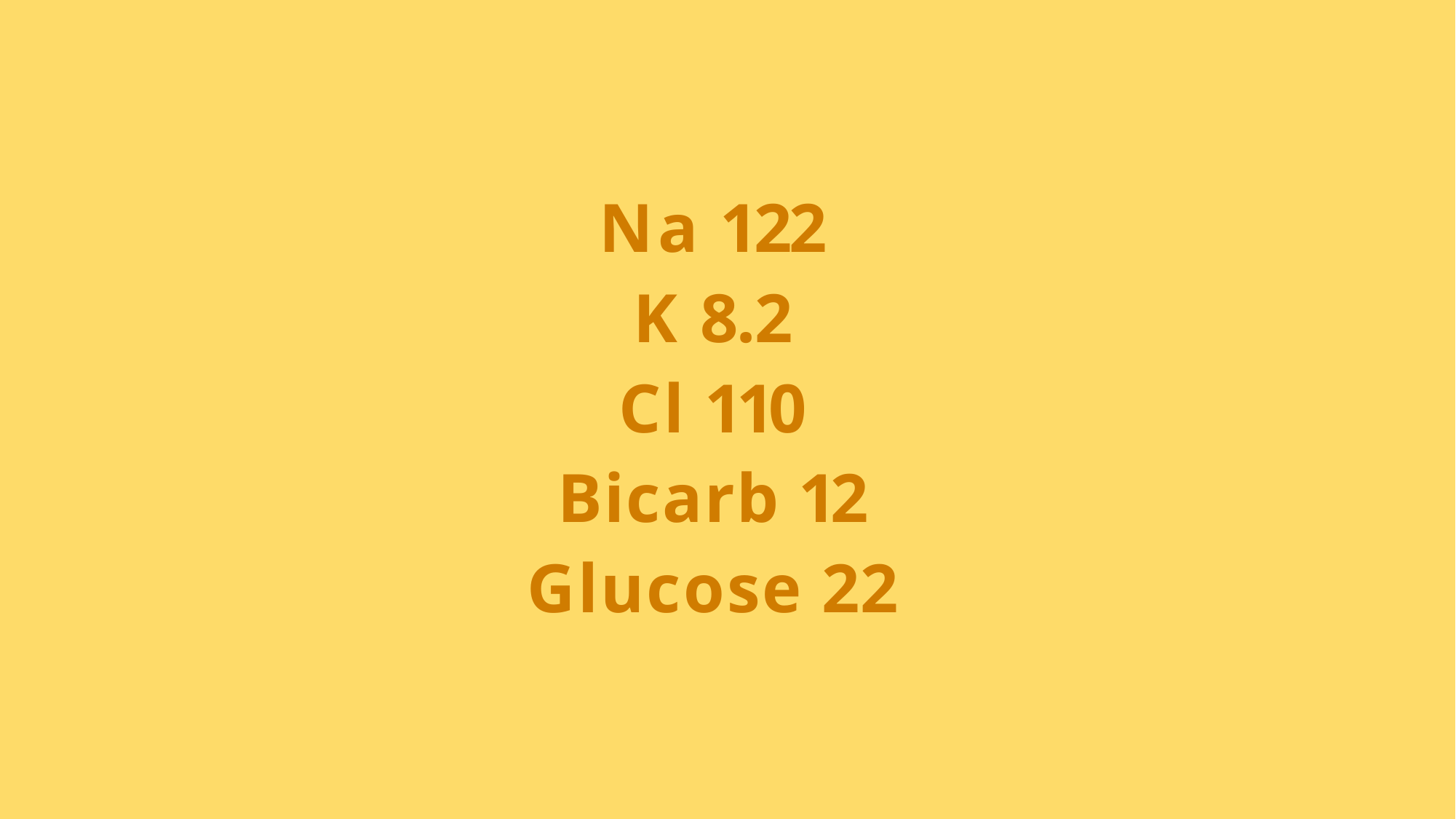

Na 122
K 8.2
Cl 110
Bicarb 12
Glucose 22

## Slide 26
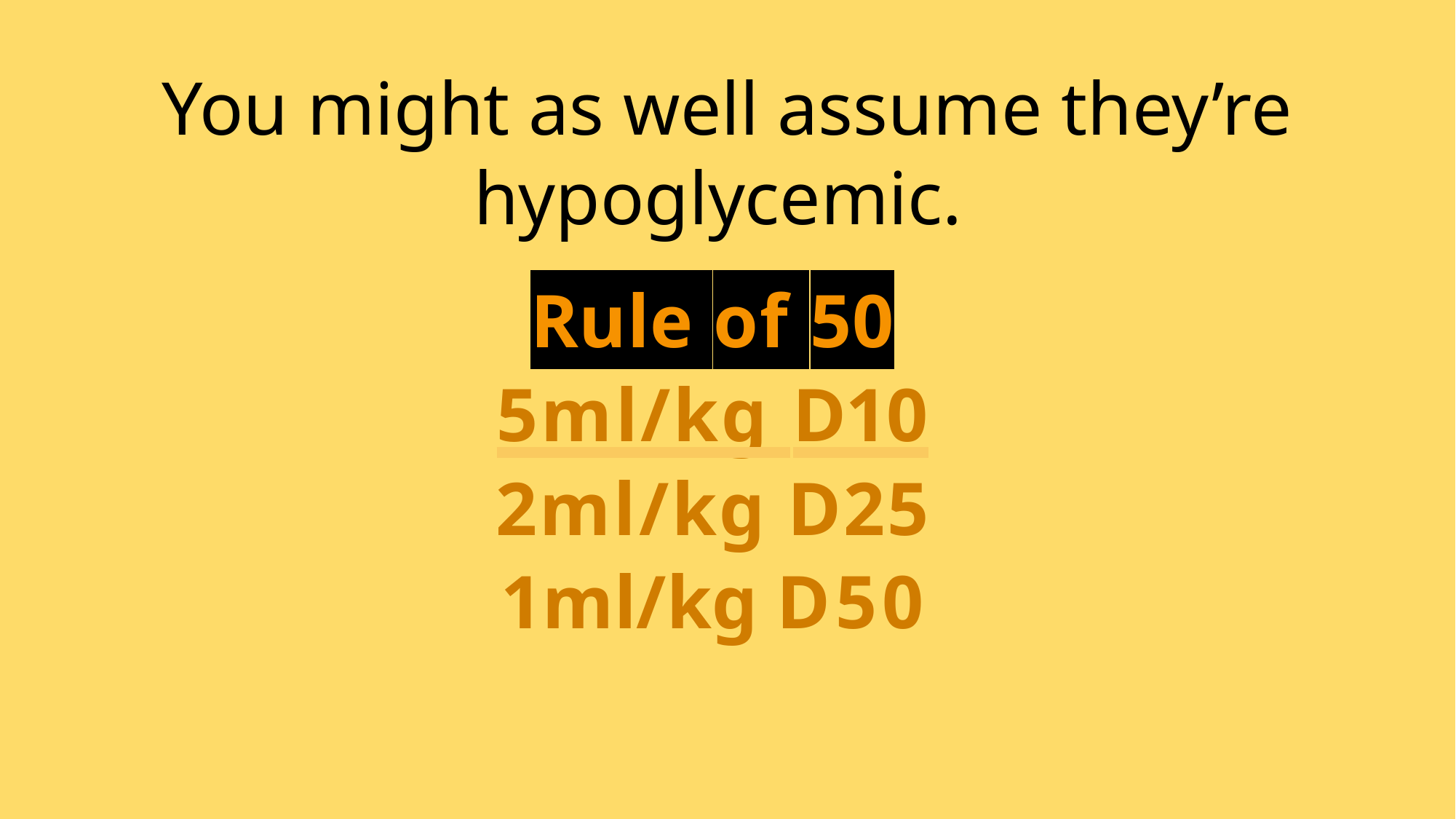

# You might as well assume they’re hypoglycemic.
Rule of 50
5ml/kg D10
2ml/kg D25
1ml/kg D50

## Slide 27
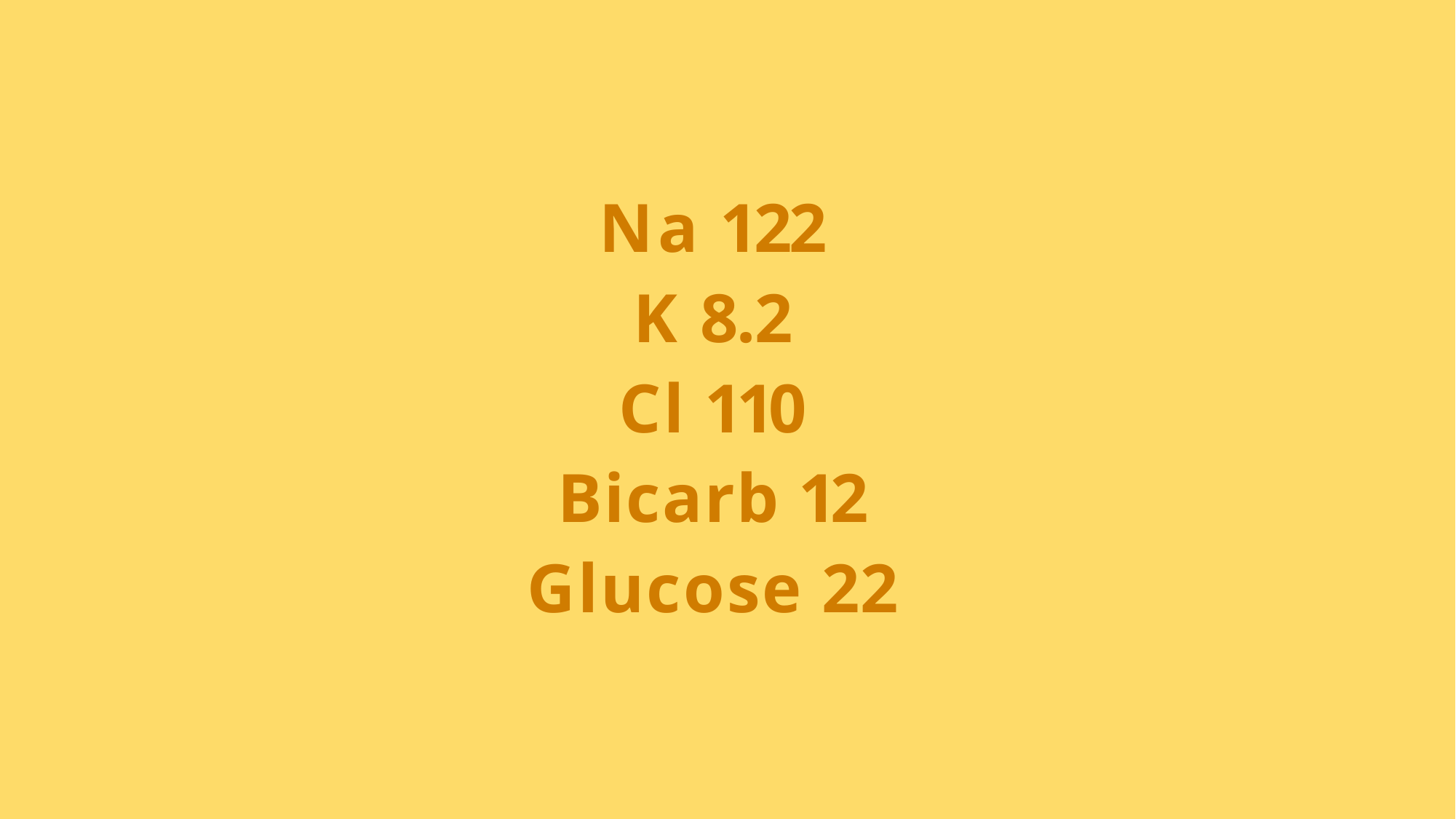

Na 122
K 8.2
Cl 110
Bicarb 12
Glucose 22

## Slide 28
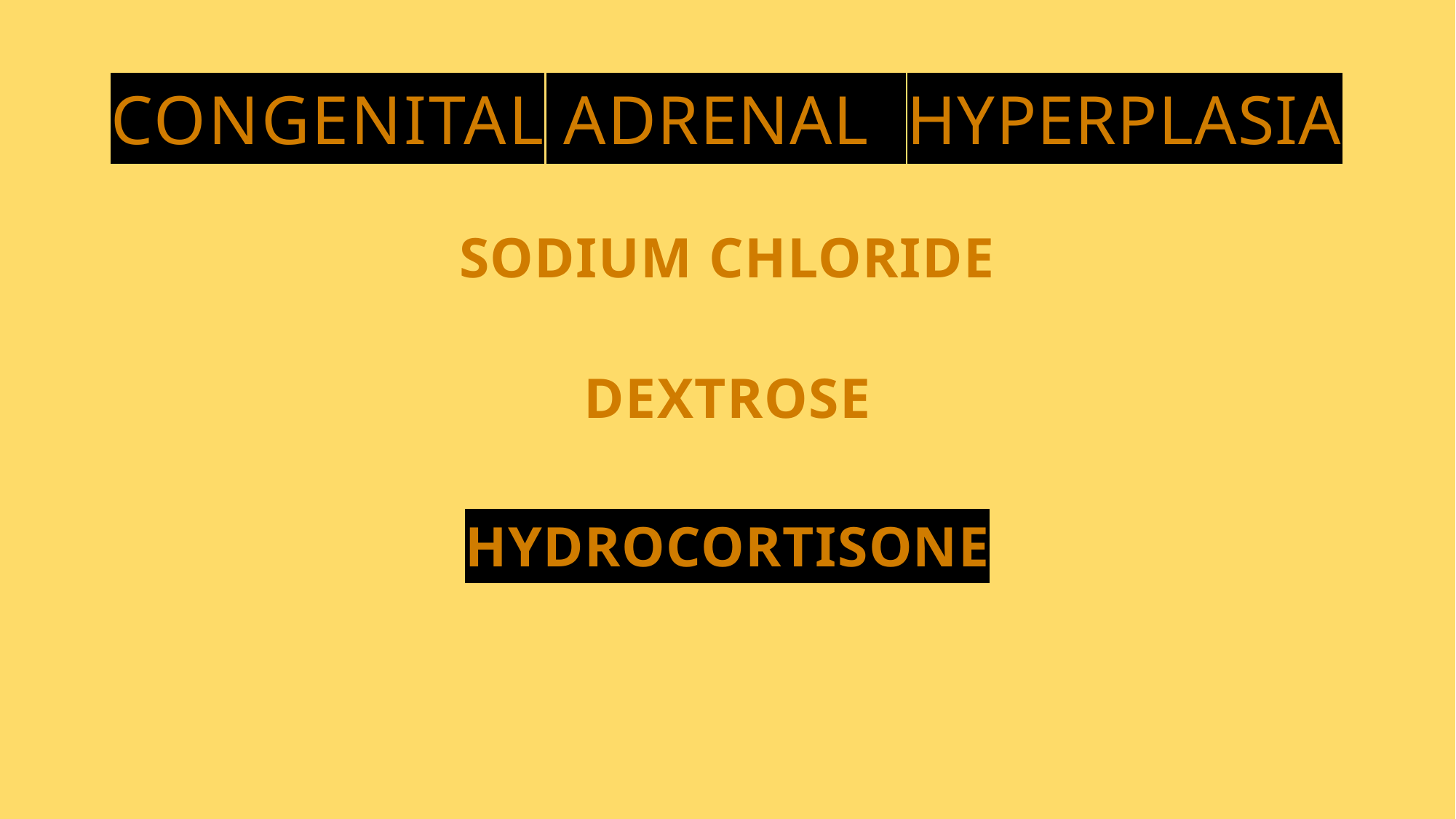

# CONGENITAL ADRENAL HYPERPLASIA
SODIUM CHLORIDE
DEXTROSE
HYDROCORTISONE

## Slide 29
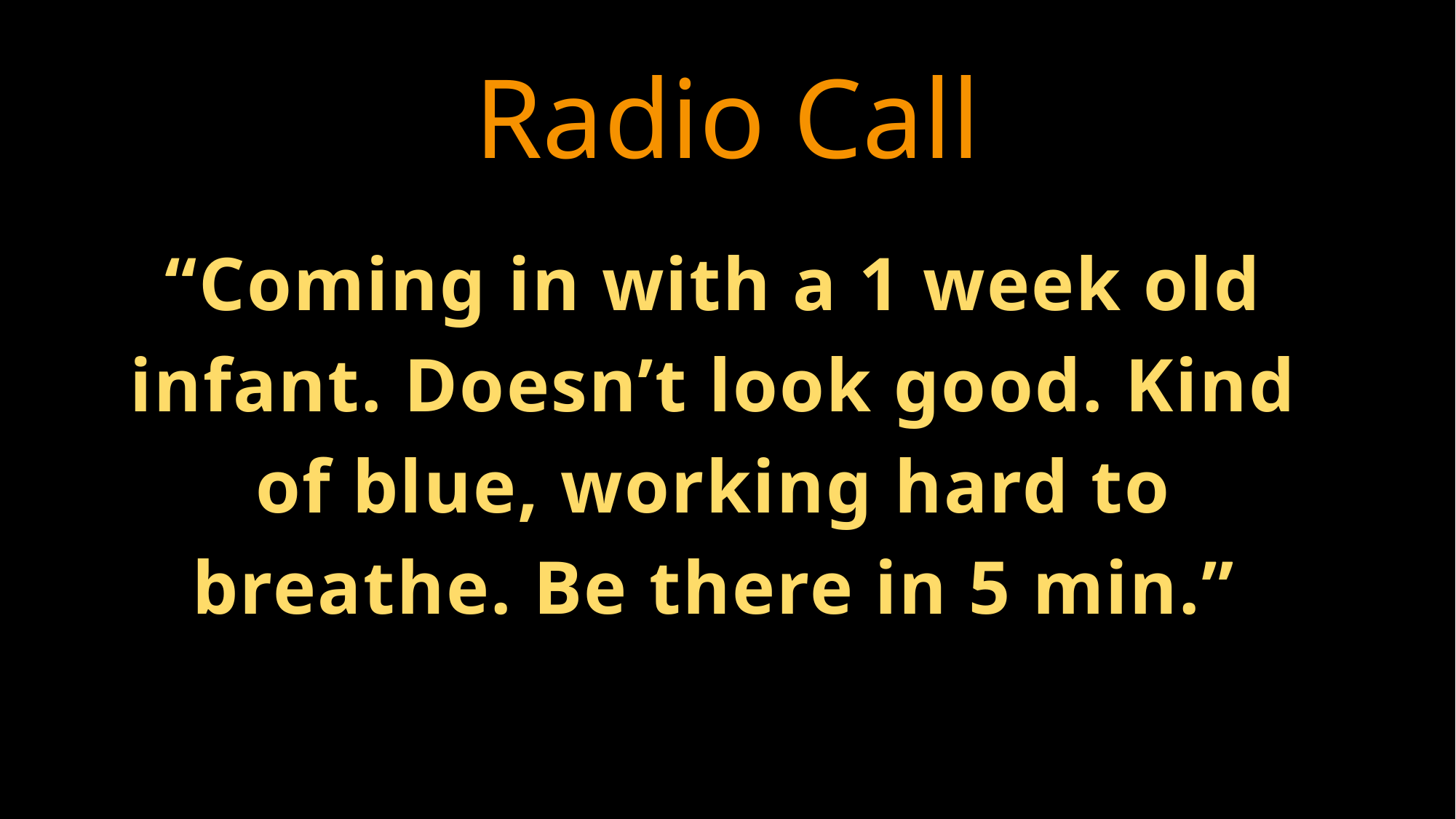

# Radio Call
“Coming in with a 1 week old infant. Doesn’t look good. Kind of blue, working hard to breathe. Be there in 5 min.”

## Slide 30
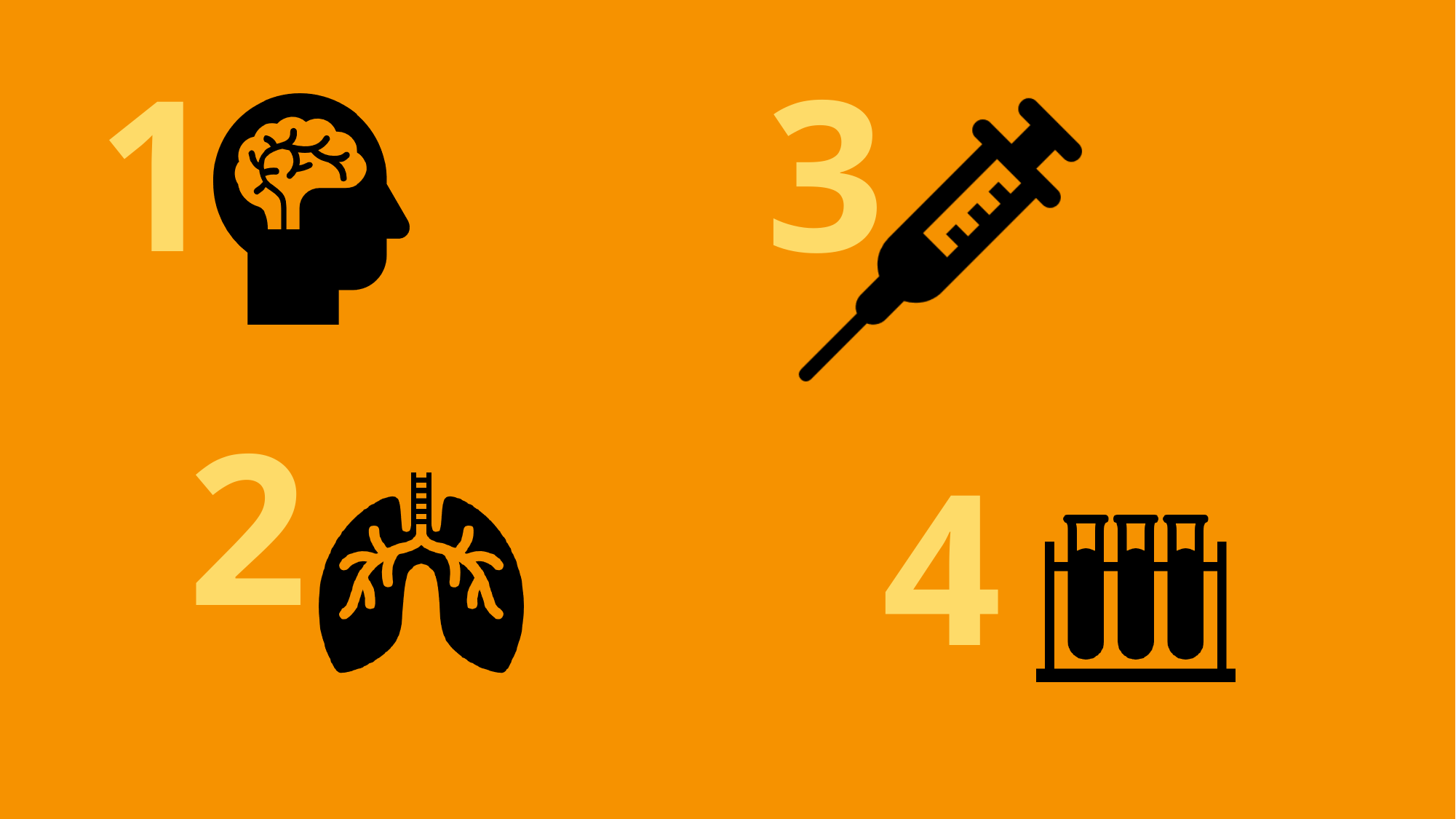

1
3
2
4

## Slide 31
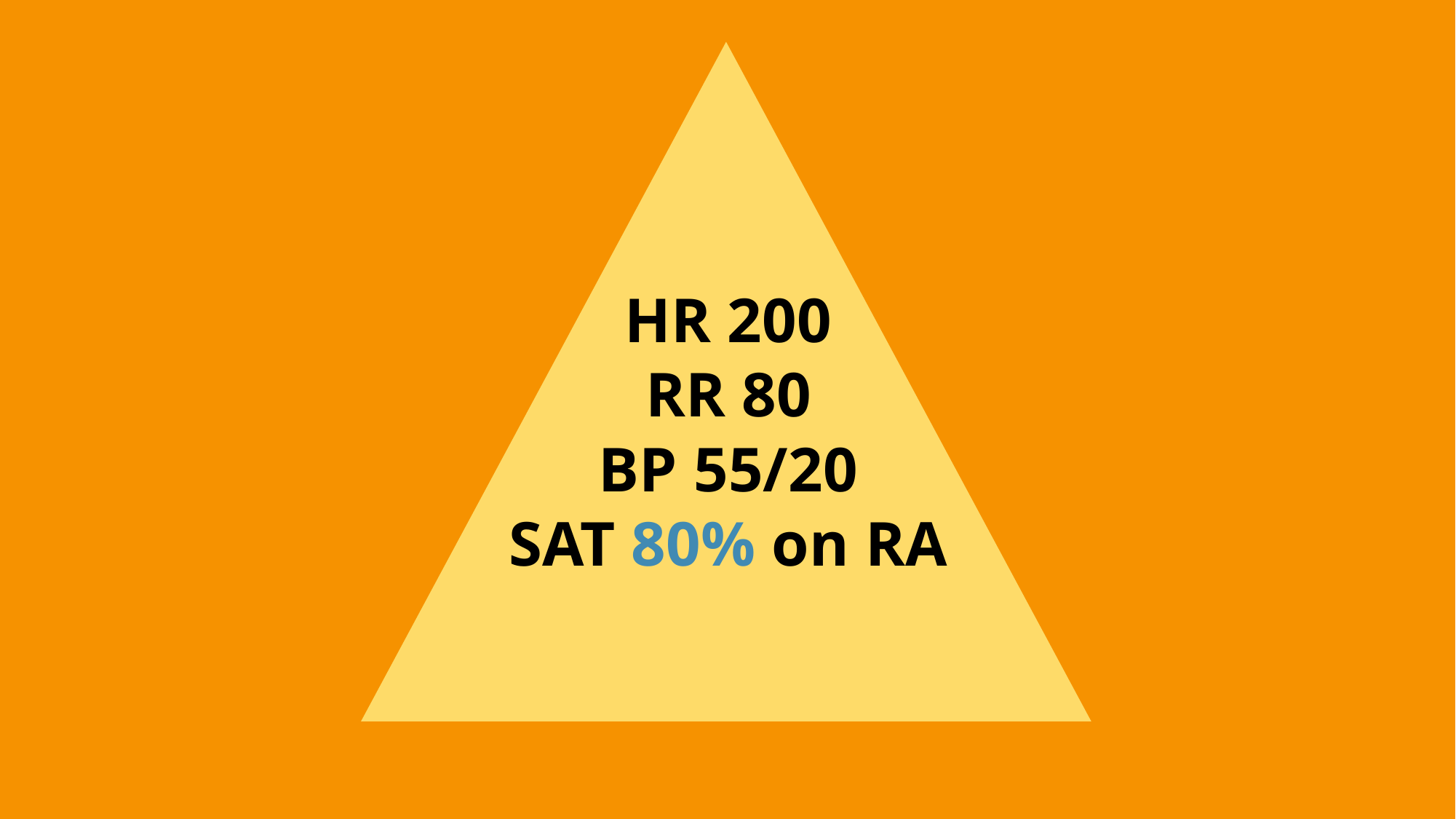

HR 200
RR 80
BP 55/20
SAT 80% on RA

## Slide 32
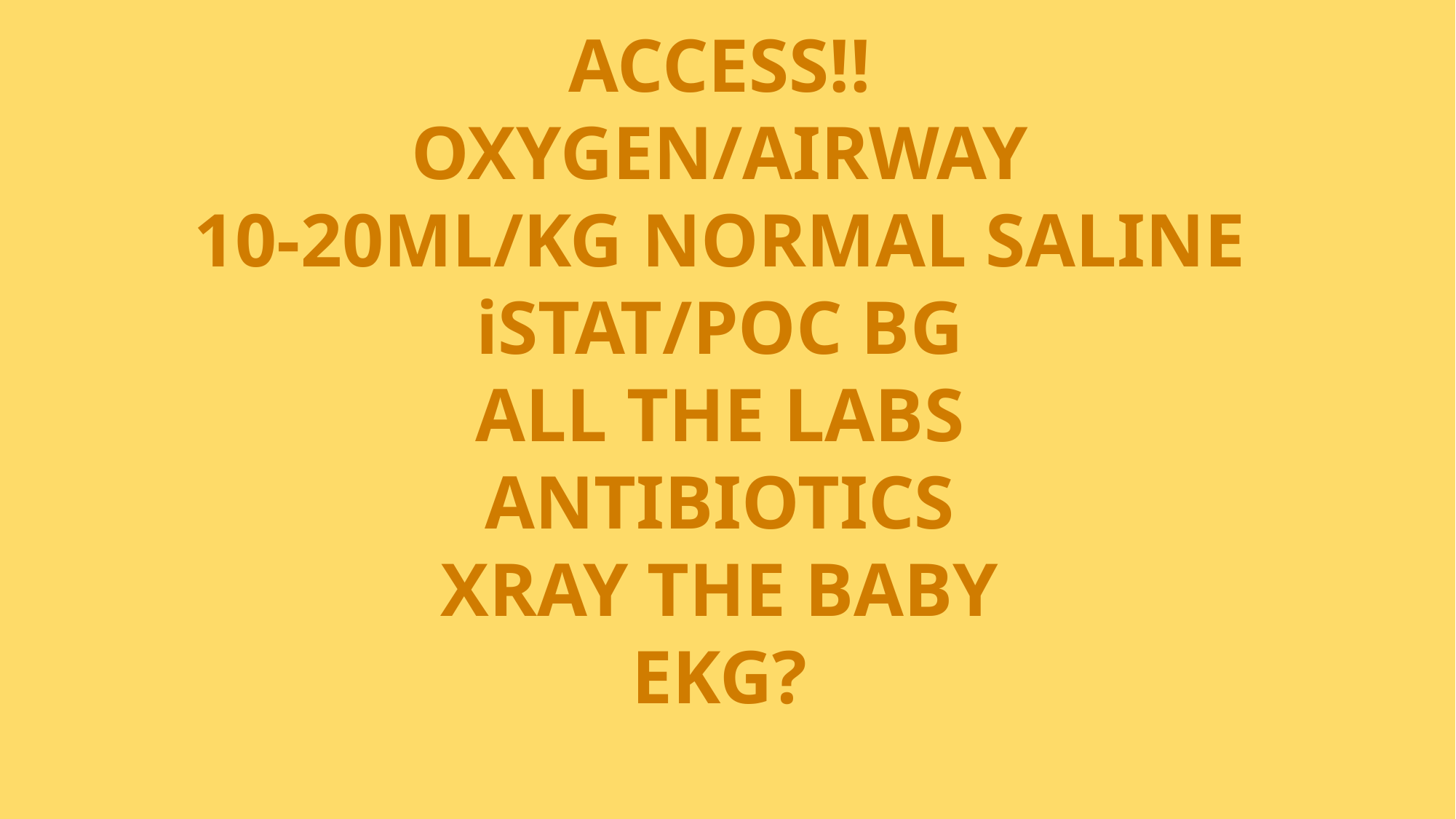

ACCESS!!
OXYGEN/AIRWAY
10-20ML/KG NORMAL SALINE
iSTAT/POC BG
ALL THE LABS
ANTIBIOTICS
XRAY THE BABY
EKG?

## Slide 33
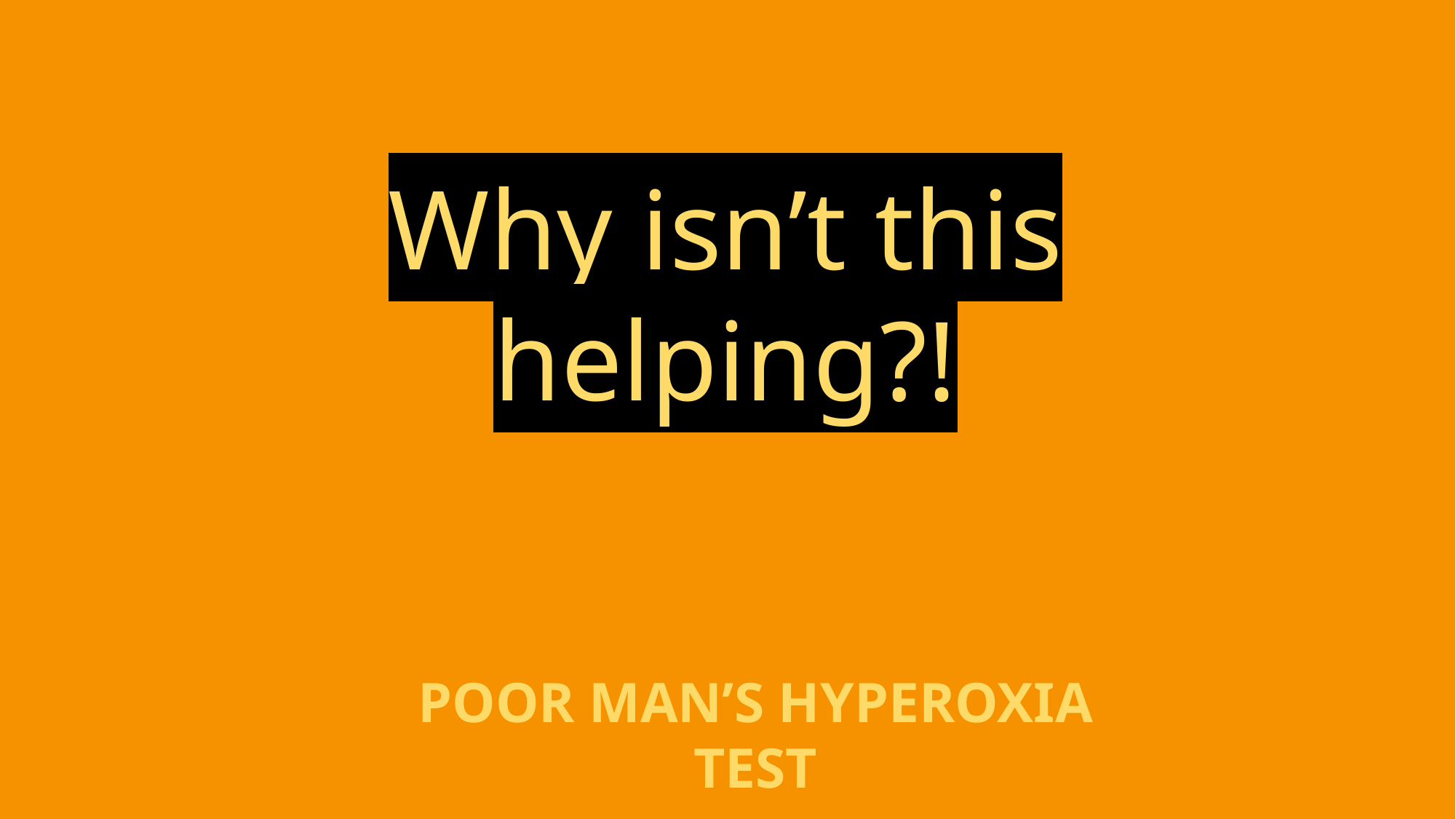

Why isn’t this helping?!
POOR MAN’S HYPEROXIA TEST

## Slide 34
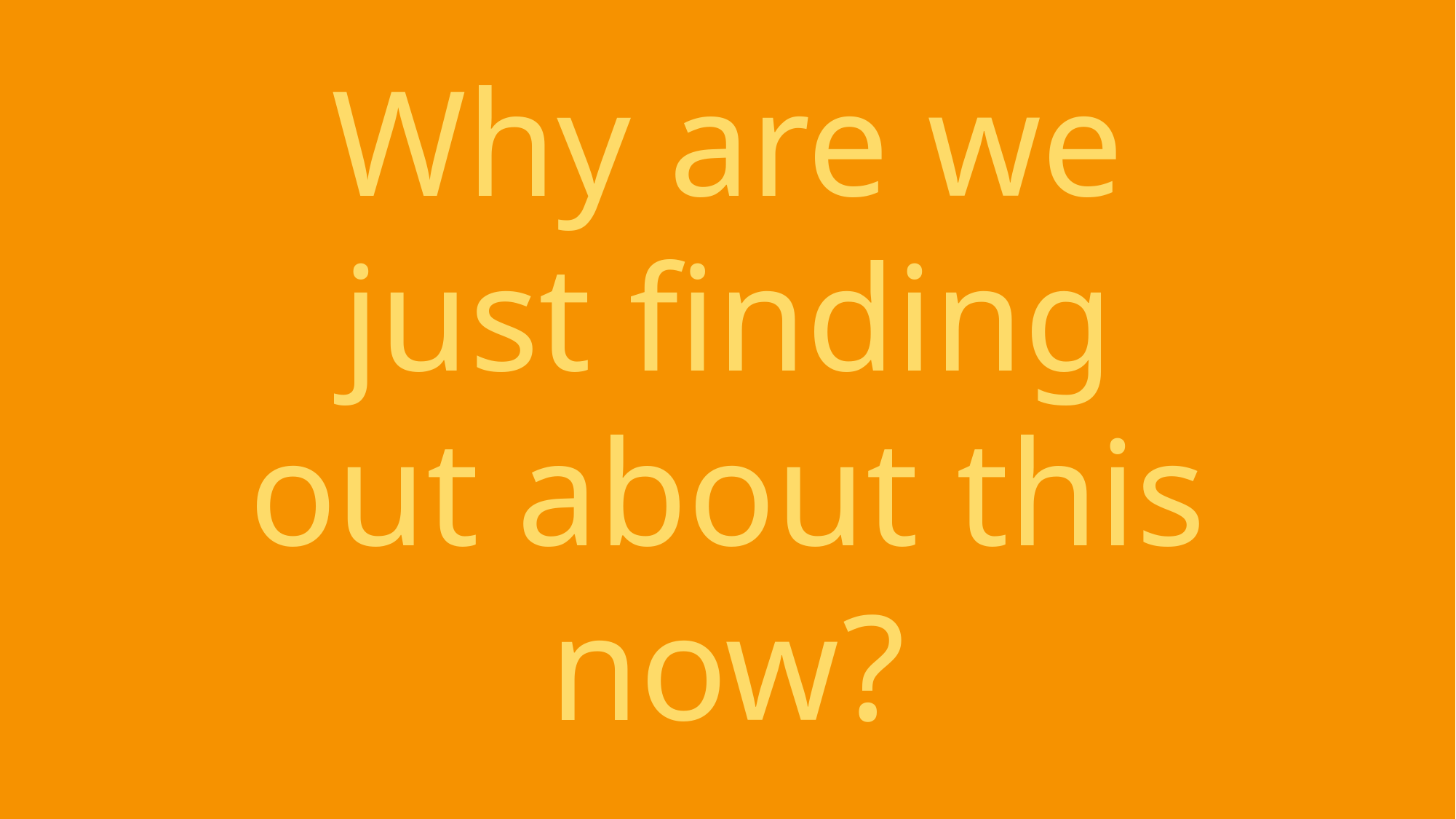

Why are we just finding out about this now?

## Slide 35
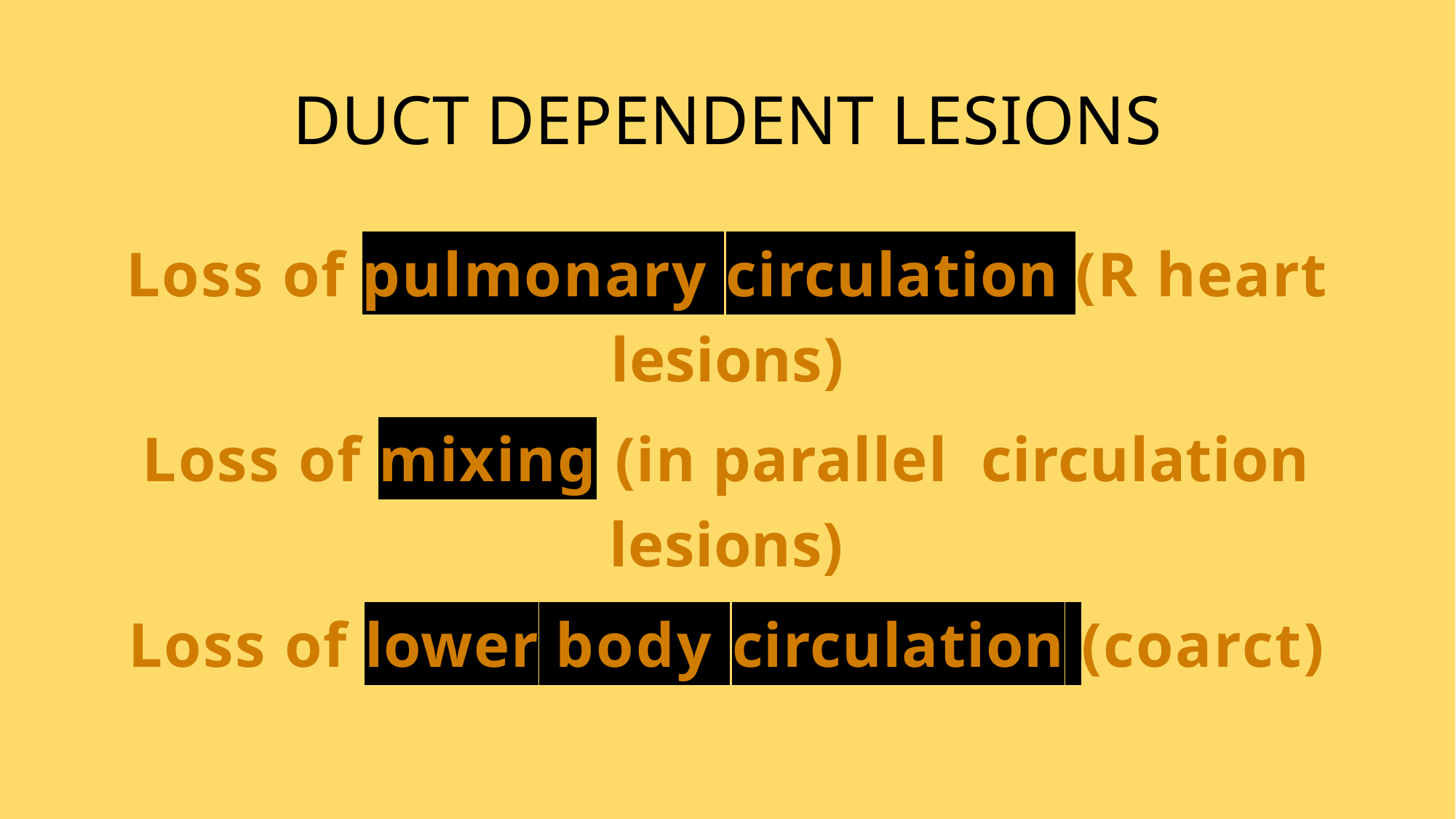

# DUCT DEPENDENT LESIONS
Loss of pulmonary circulation (R heart lesions)
Loss of mixing (in parallel circulation lesions)
Loss of lower body circulation (coarct)

## Slide 36
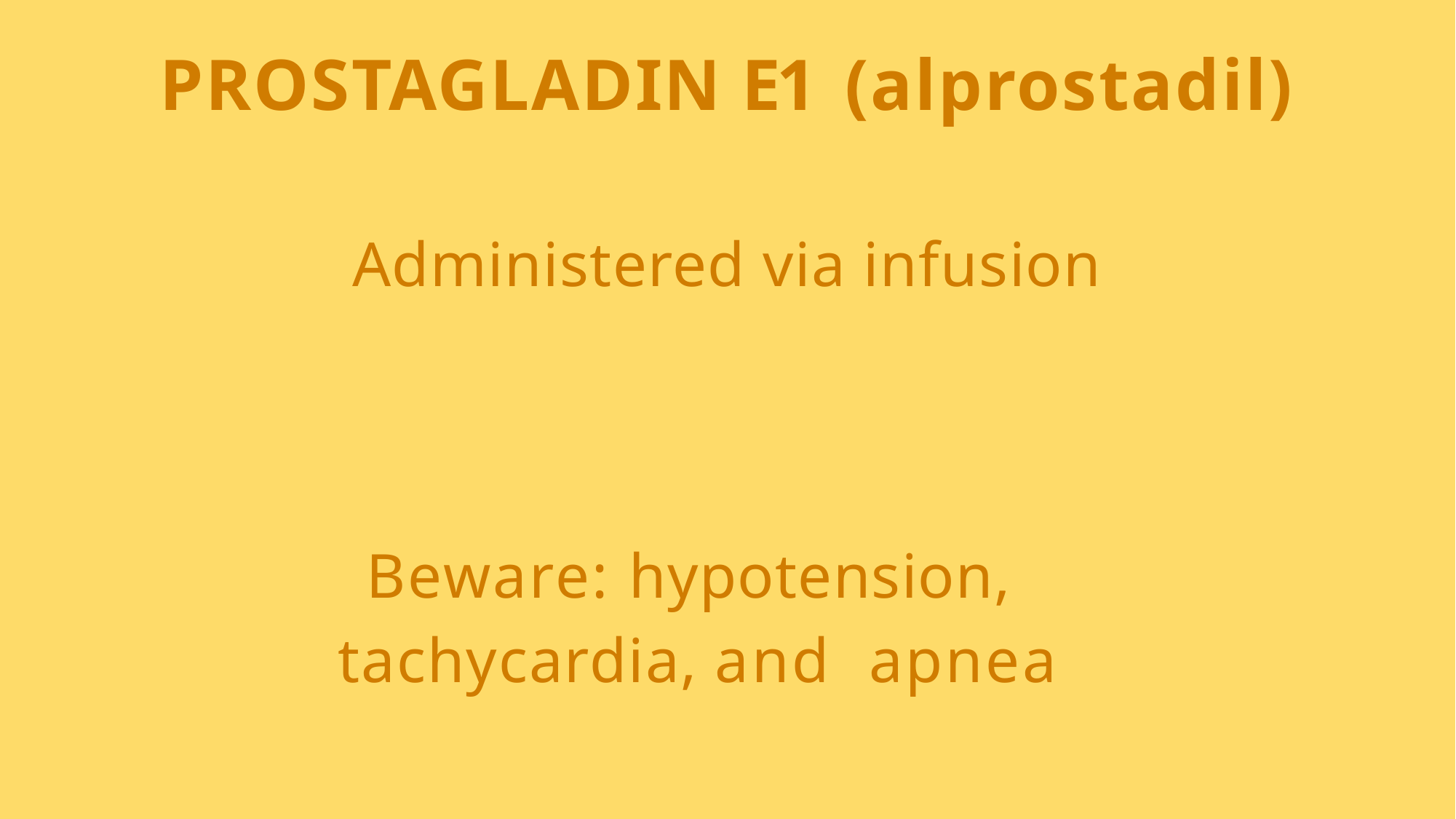

# PROSTAGLADIN E1 (alprostadil)
Administered via infusion
Beware: hypotension, tachycardia, and apnea

## Slide 37
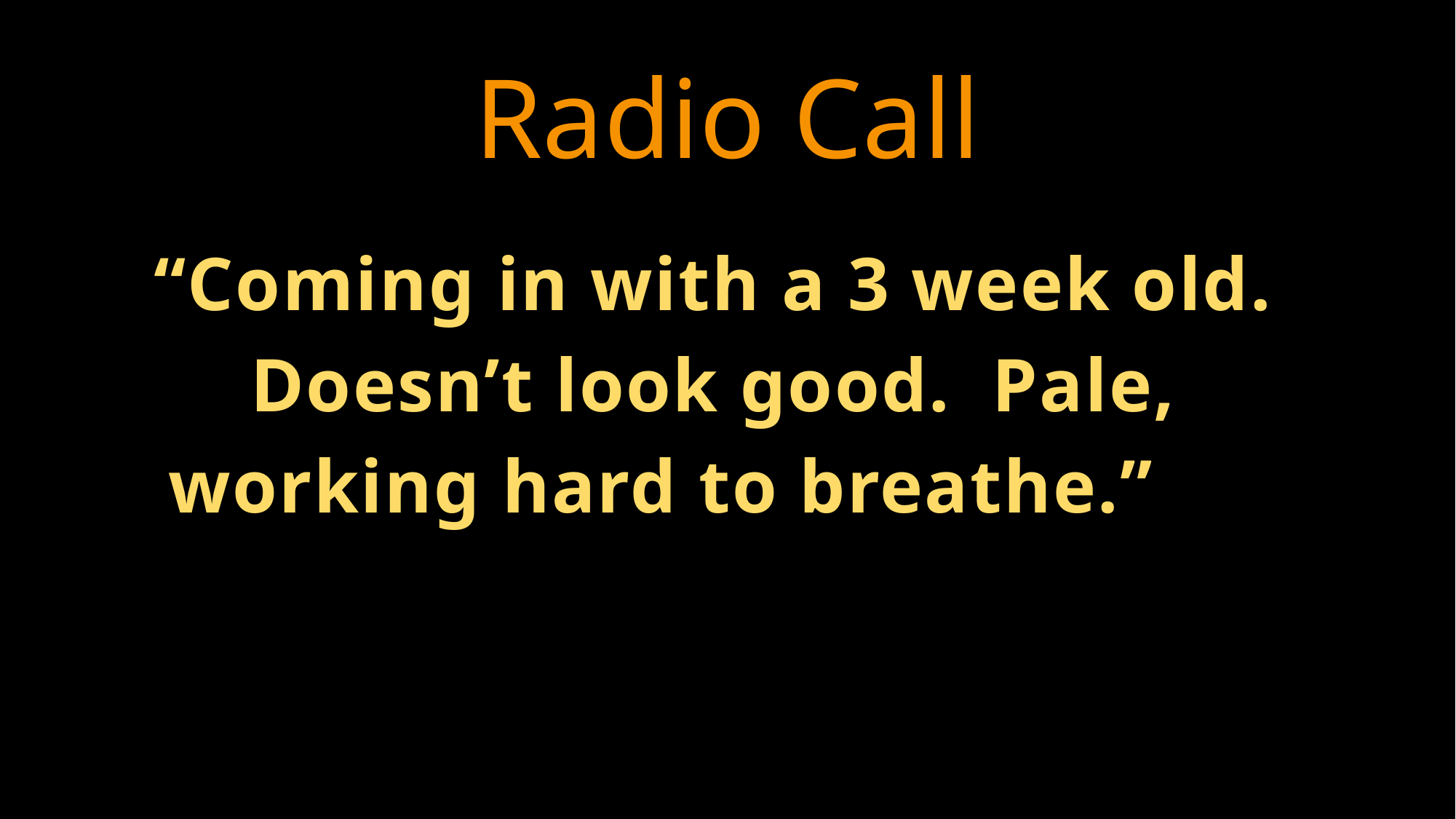

# Radio Call
“Coming in with a 3 week old. Doesn’t look good. Pale, working hard to breathe.”

## Slide 38
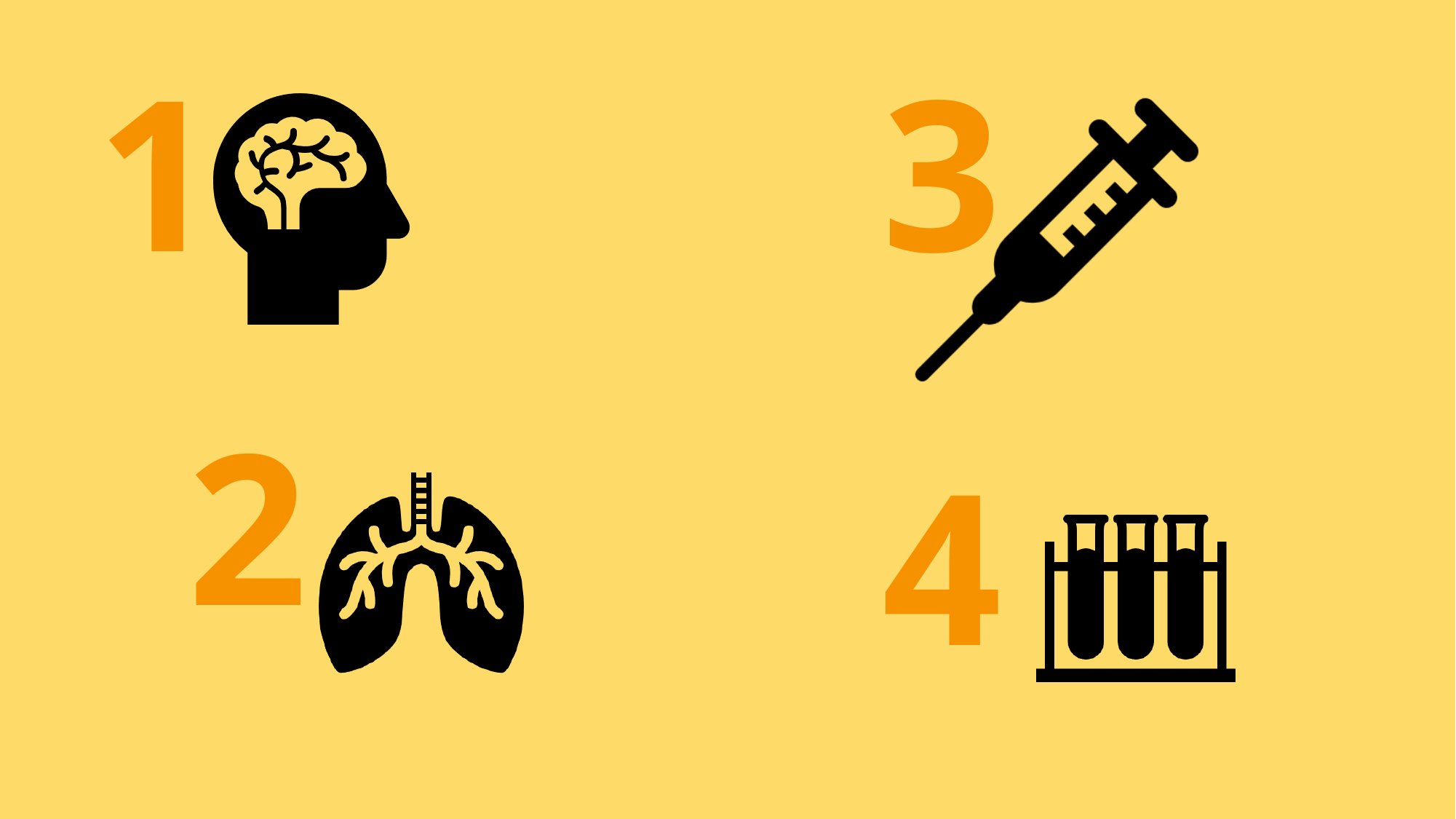

1
3
2
4

## Slide 39
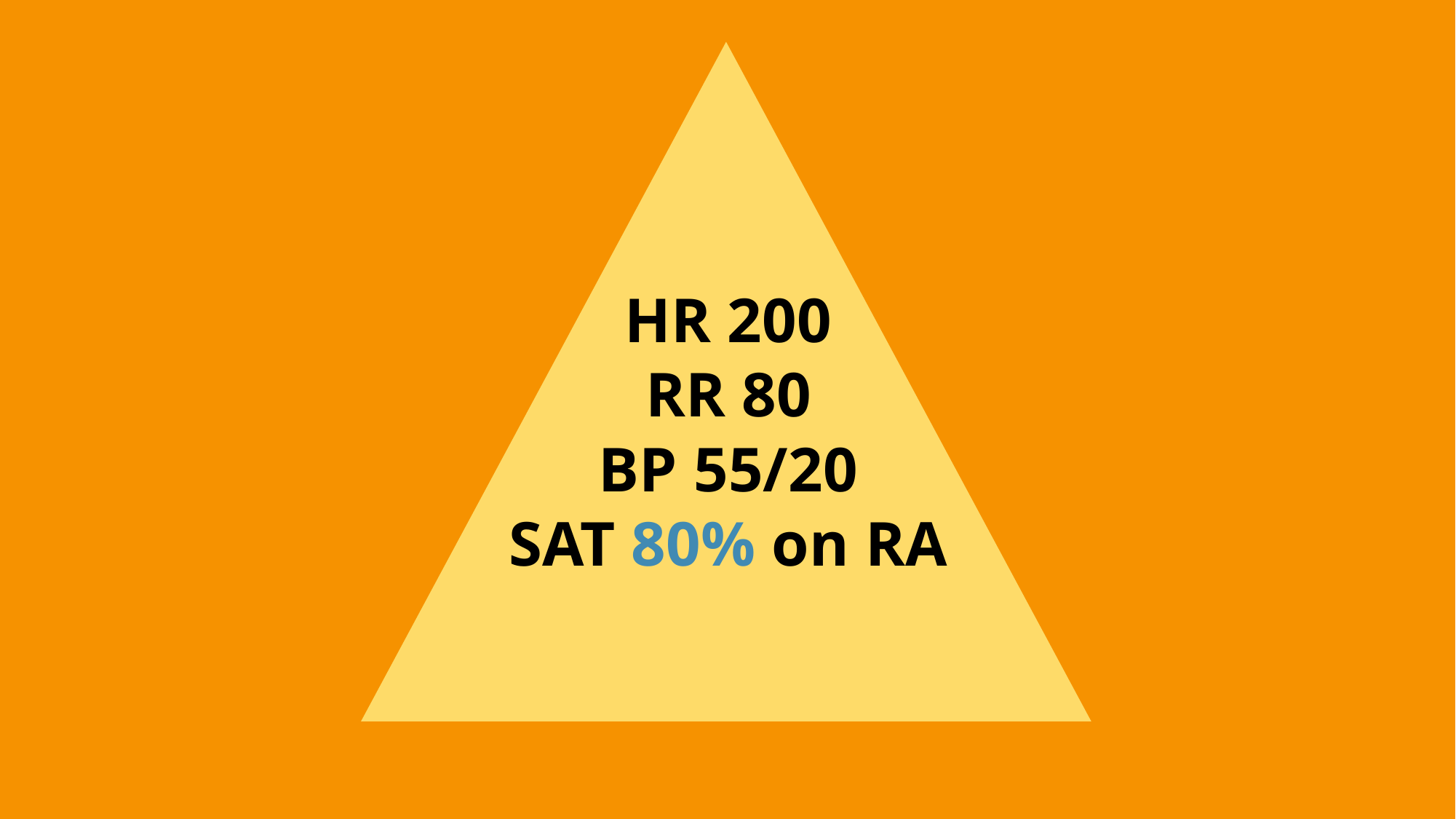

HR 200
RR 80
BP 55/20
SAT 80% on RA

## Slide 40
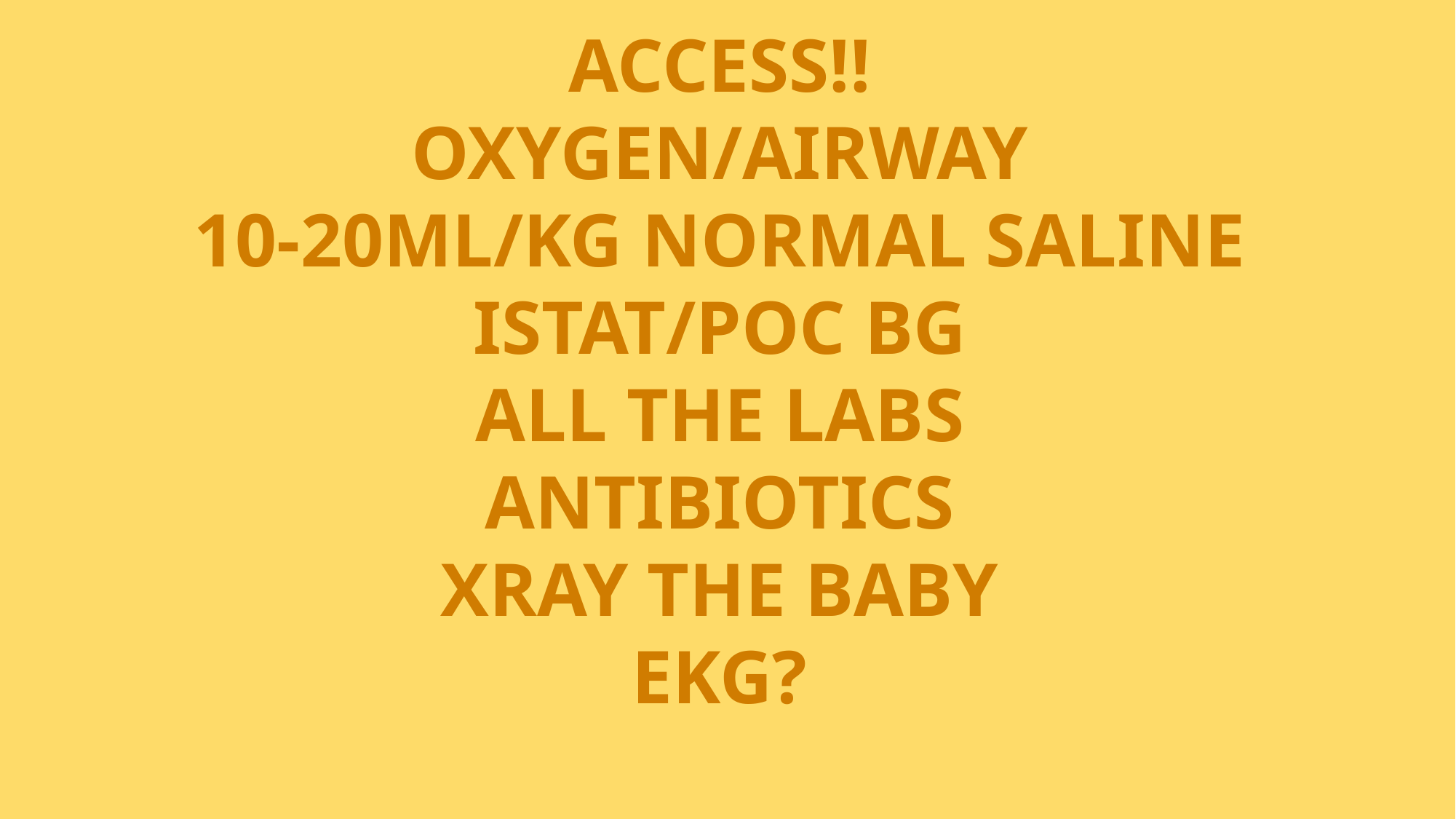

ACCESS!!
OXYGEN/AIRWAY
10-20ML/KG NORMAL SALINE
ISTAT/POC BG
ALL THE LABS
ANTIBIOTICS
XRAY THE BABY
EKG?

## Slide 41
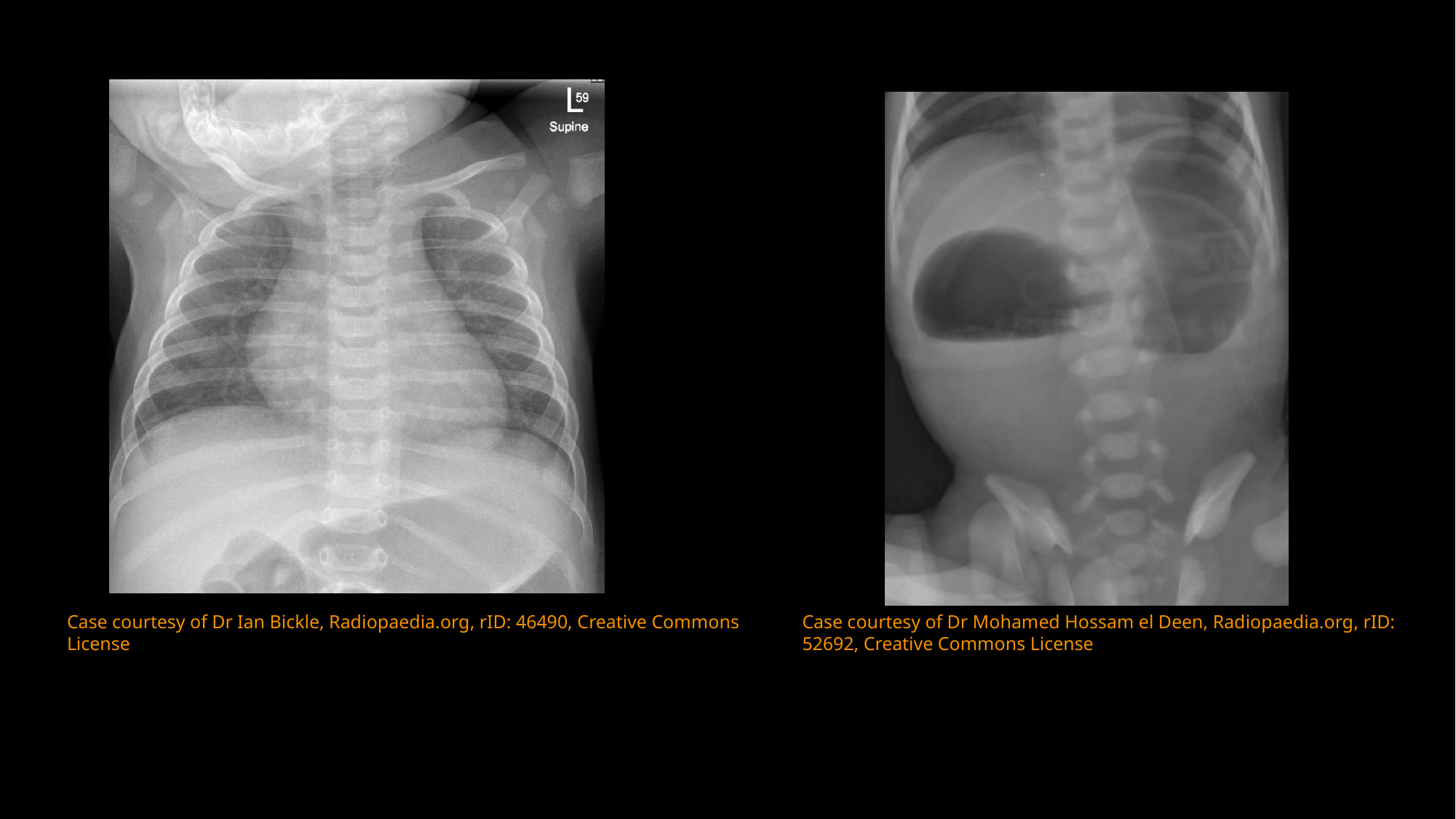

Case courtesy of Dr Mohamed Hossam el Deen, Radiopaedia.org, rID: 52692, Creative Commons License
Case courtesy of Dr Ian Bickle, Radiopaedia.org, rID: 46490, Creative Commons License

## Slide 42
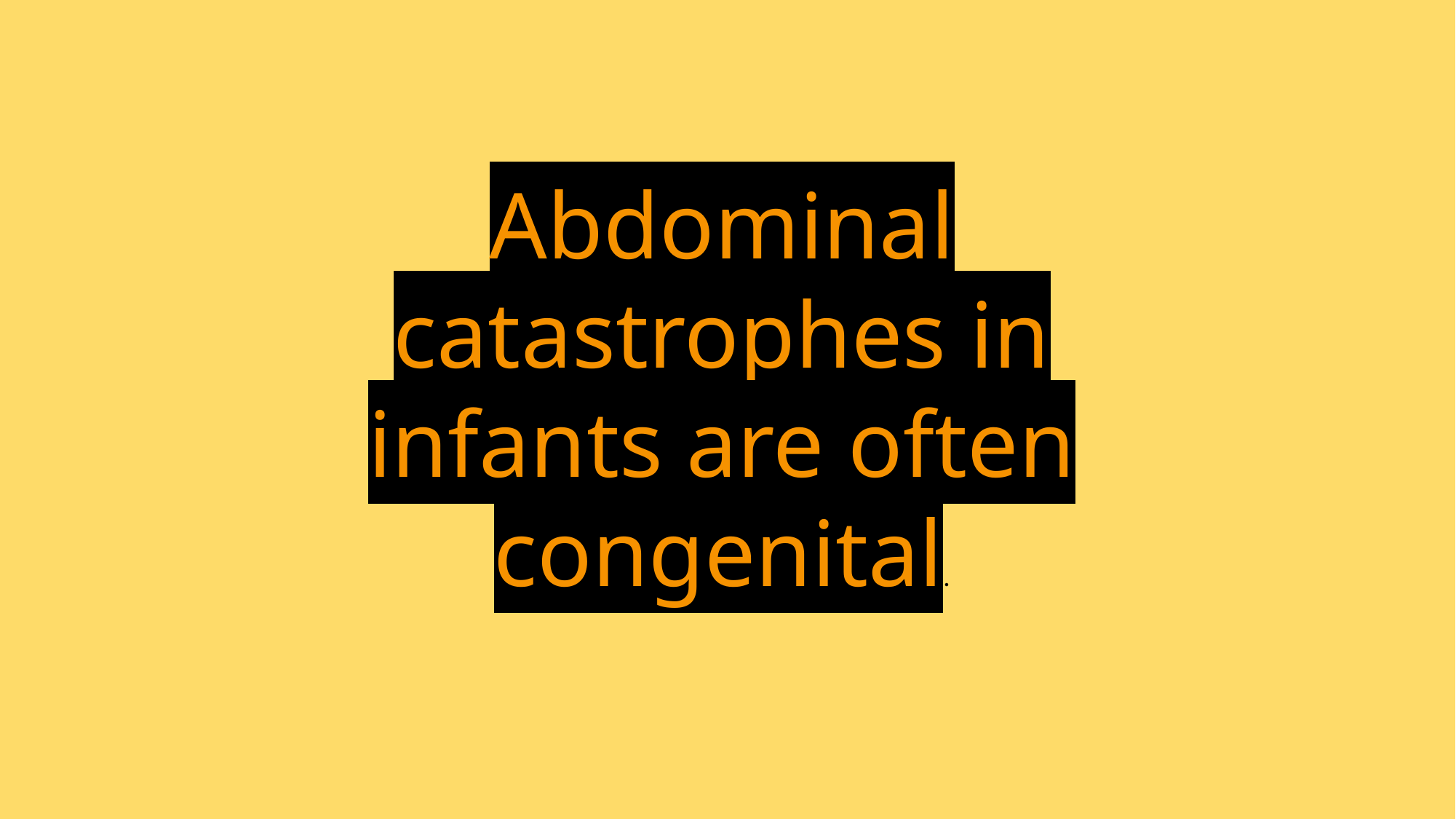

Abdominal catastrophes in infants are often congenital.

## Slide 43
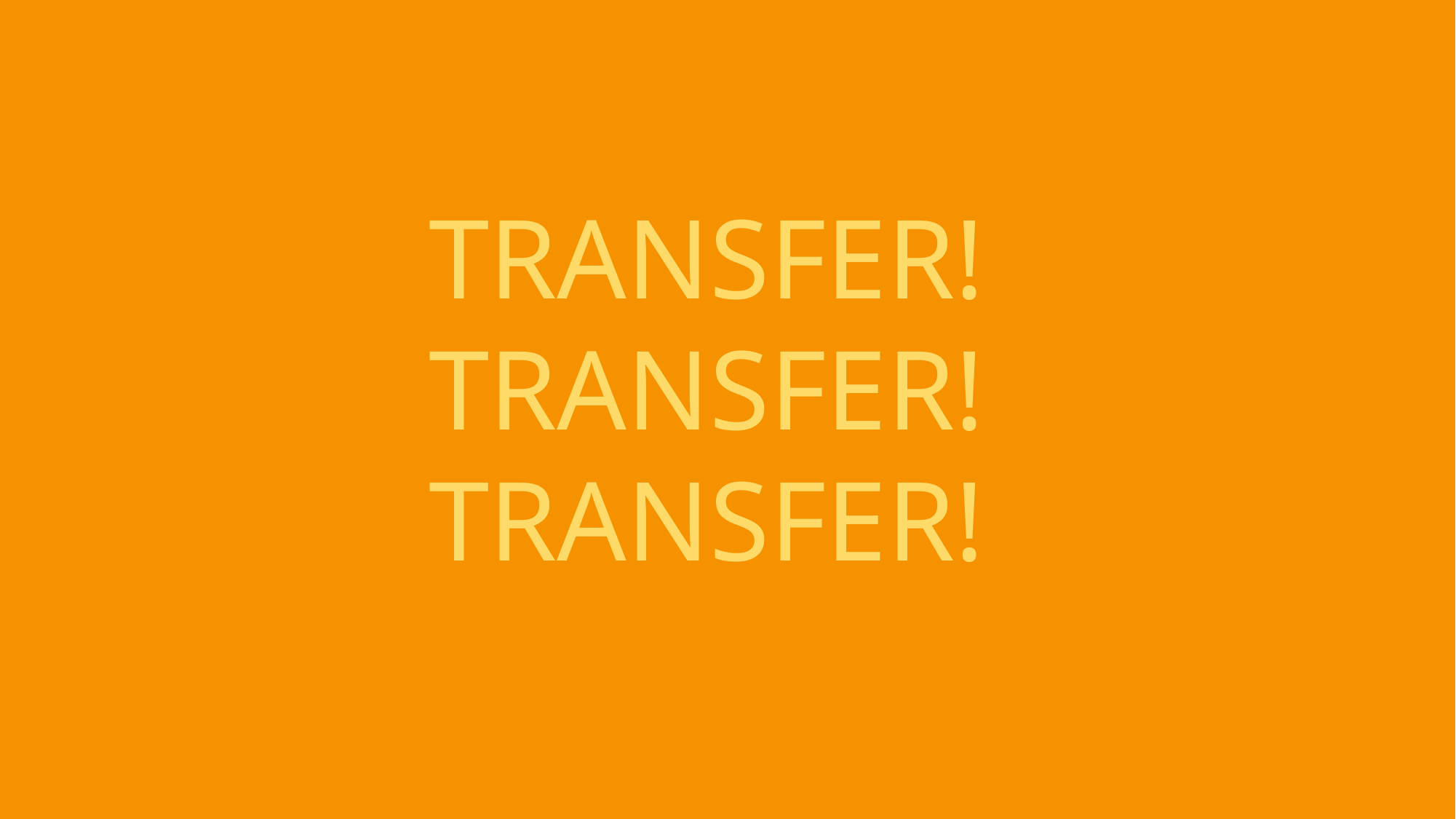

TRANSFER!
TRANSFER!
TRANSFER!

## Slide 44
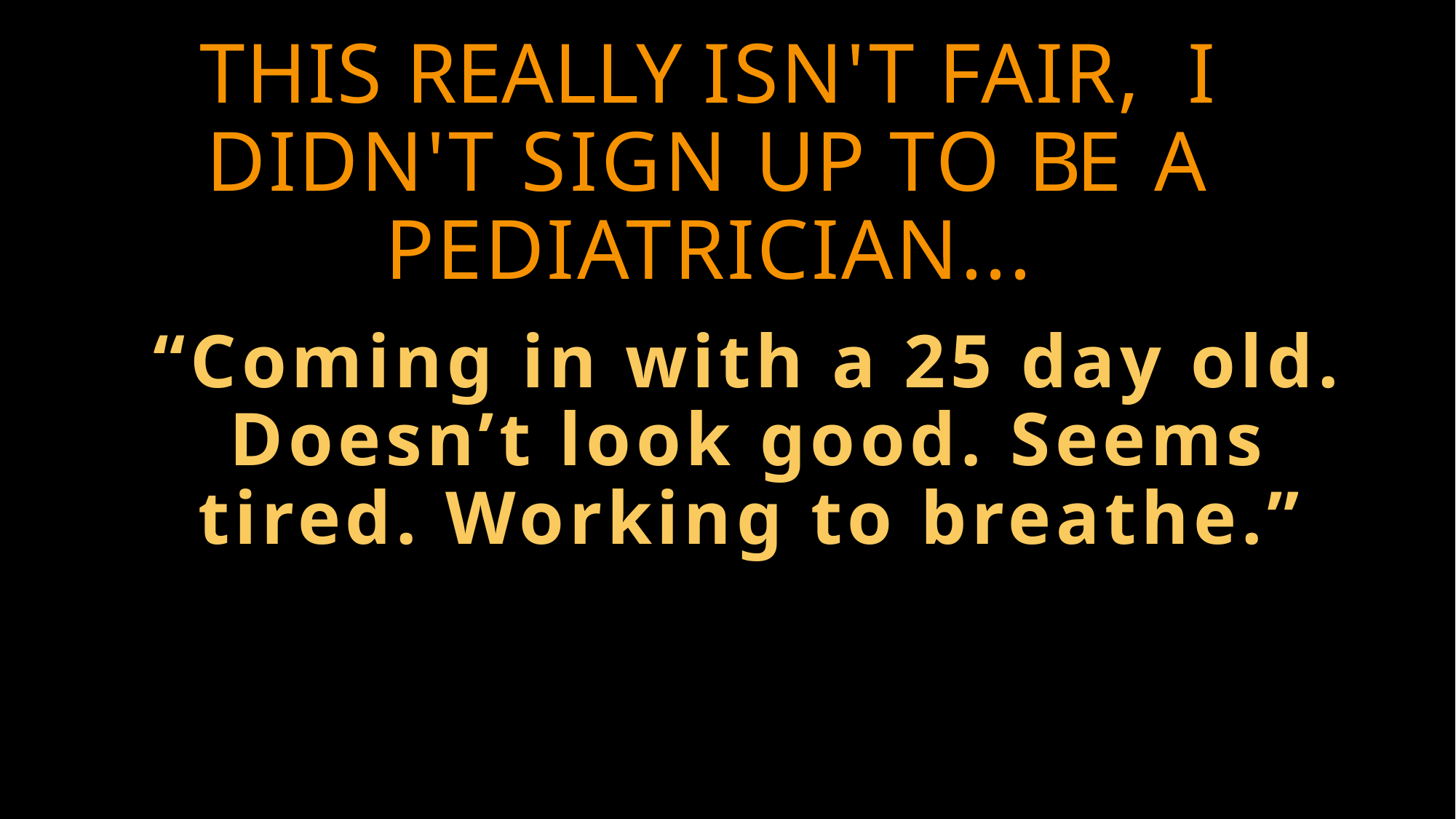

# THIS REALLY ISN'T FAIR, I DIDN'T SIGN UP TO BE A PEDIATRICIAN...
“Coming in with a 25 day old. Doesn’t look good. Seems tired. Working to breathe.”

## Slide 45
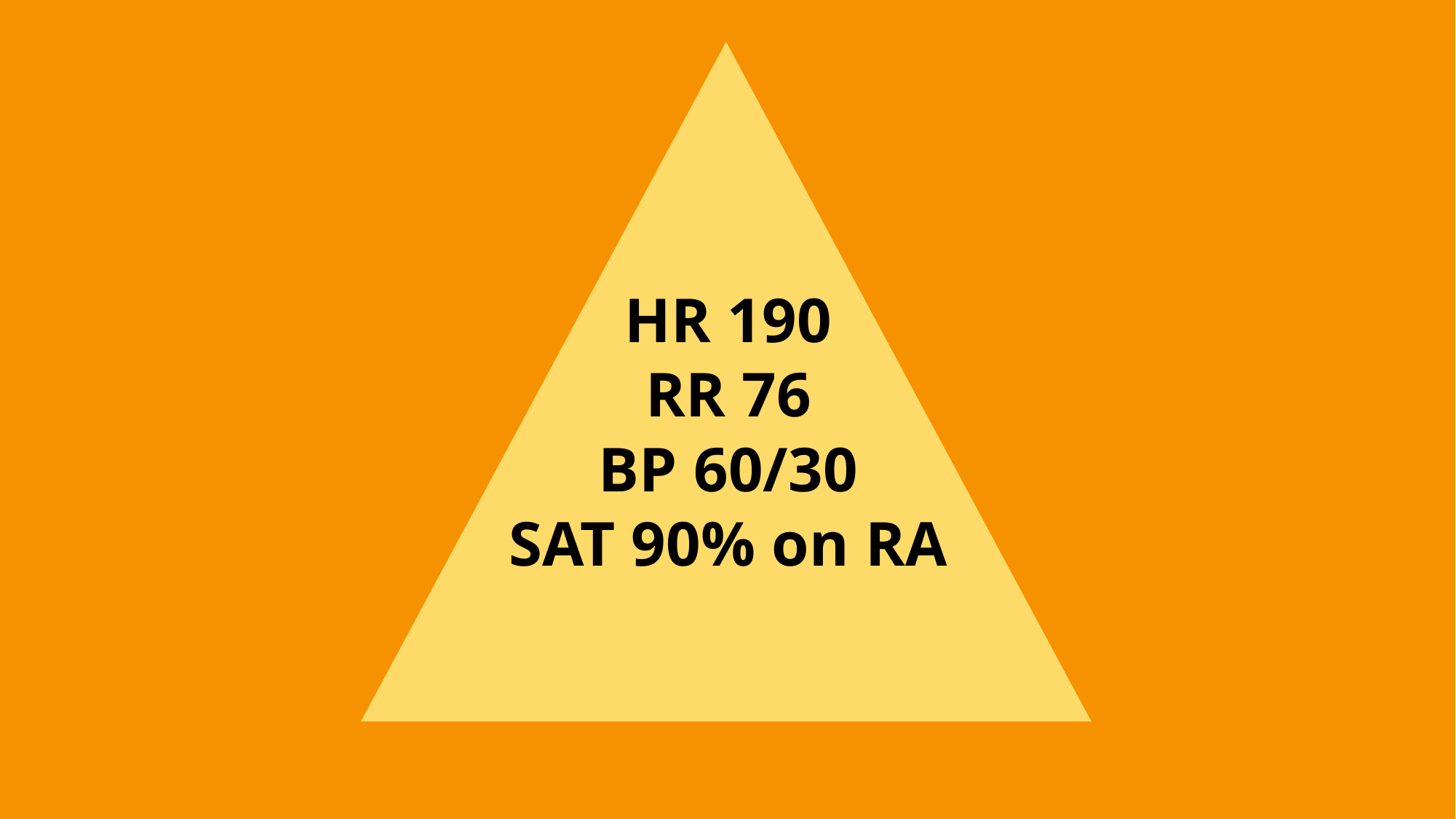

HR 190
RR 76
BP 60/30
SAT 90% on RA

## Slide 46
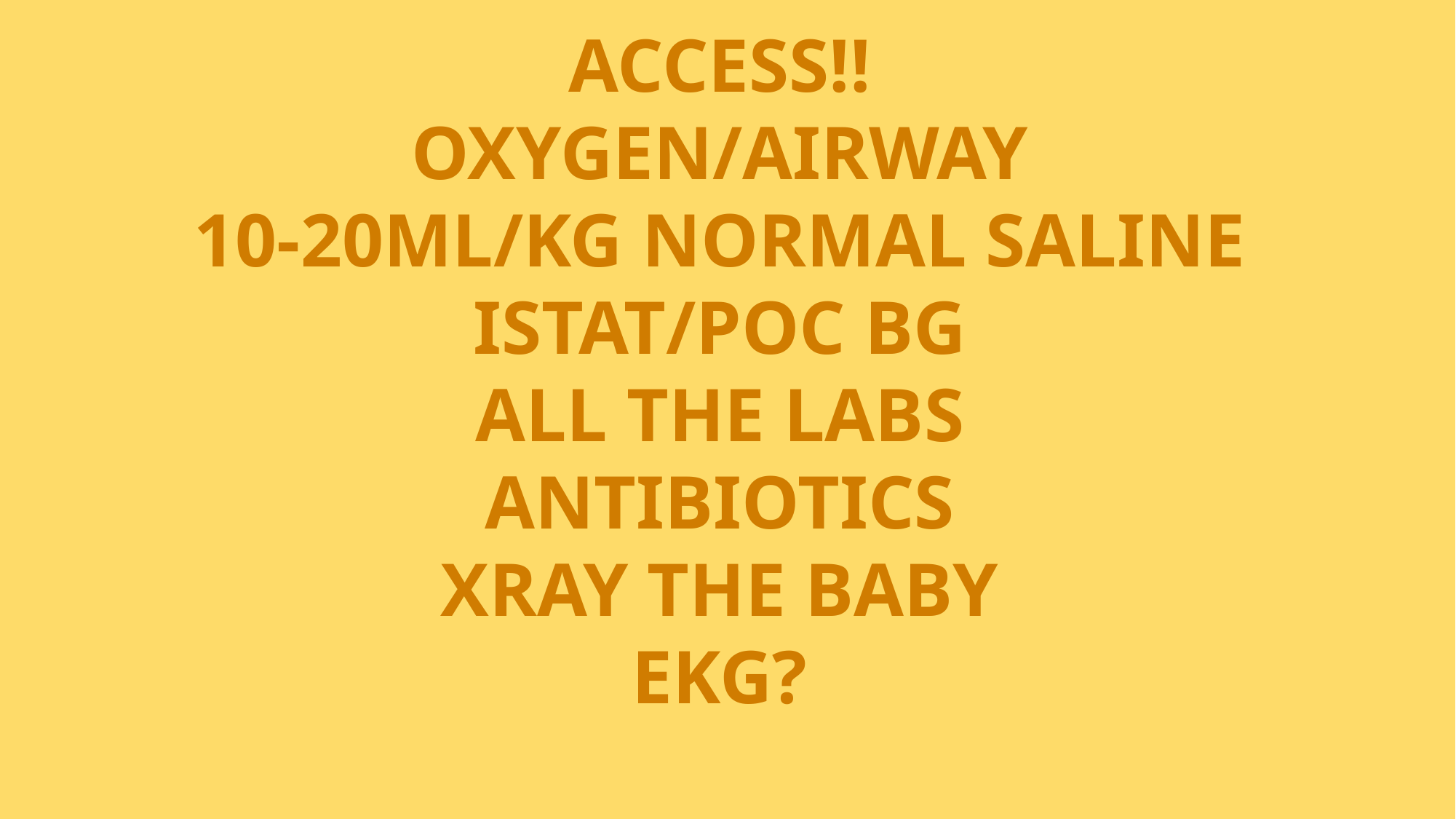

ACCESS!!
OXYGEN/AIRWAY
10-20ML/KG NORMAL SALINE
ISTAT/POC BG
ALL THE LABS
ANTIBIOTICS
XRAY THE BABY
EKG?

## Slide 47
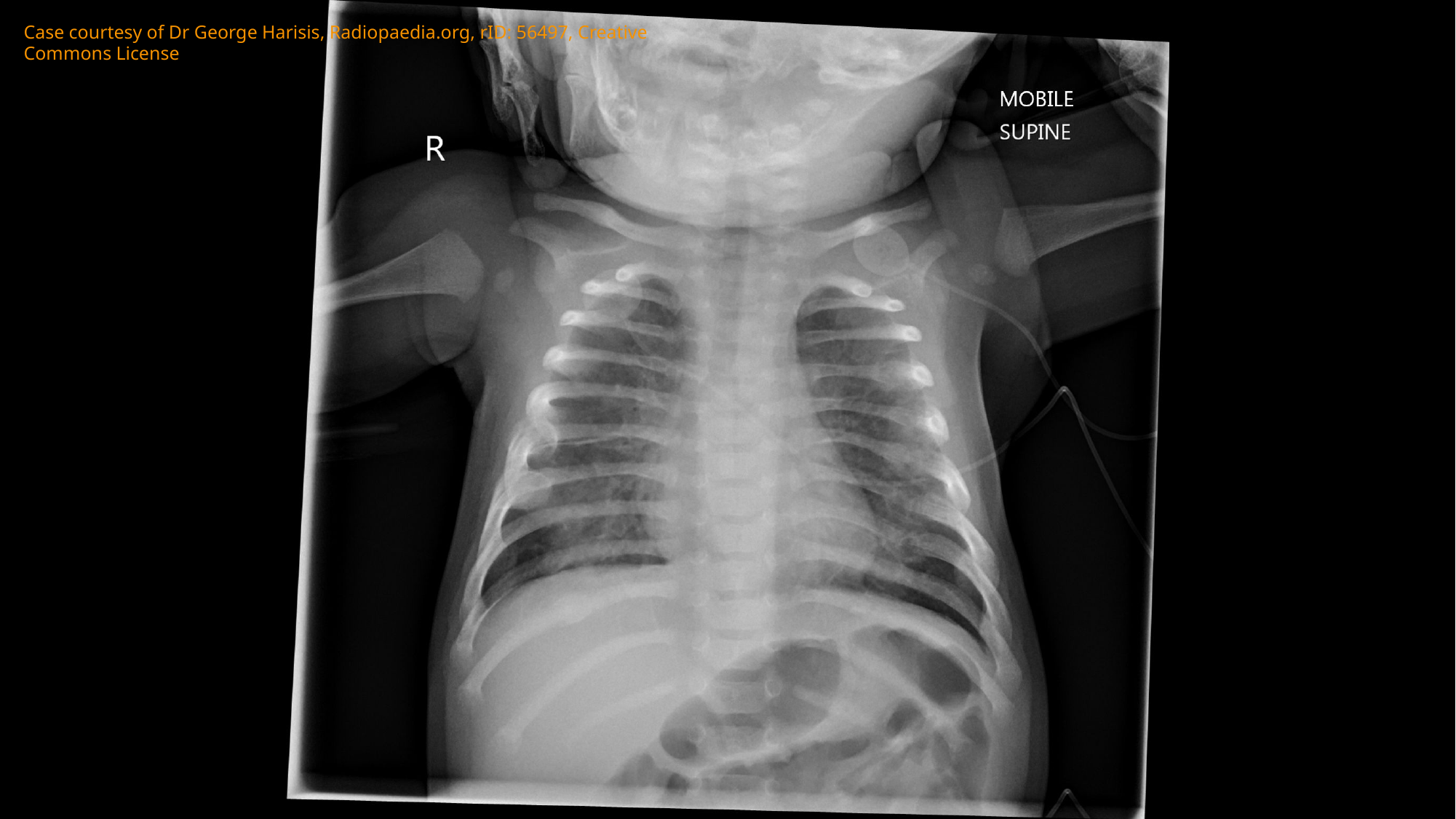

Case courtesy of Dr George Harisis, Radiopaedia.org, rID: 56497, Creative Commons License

## Slide 48
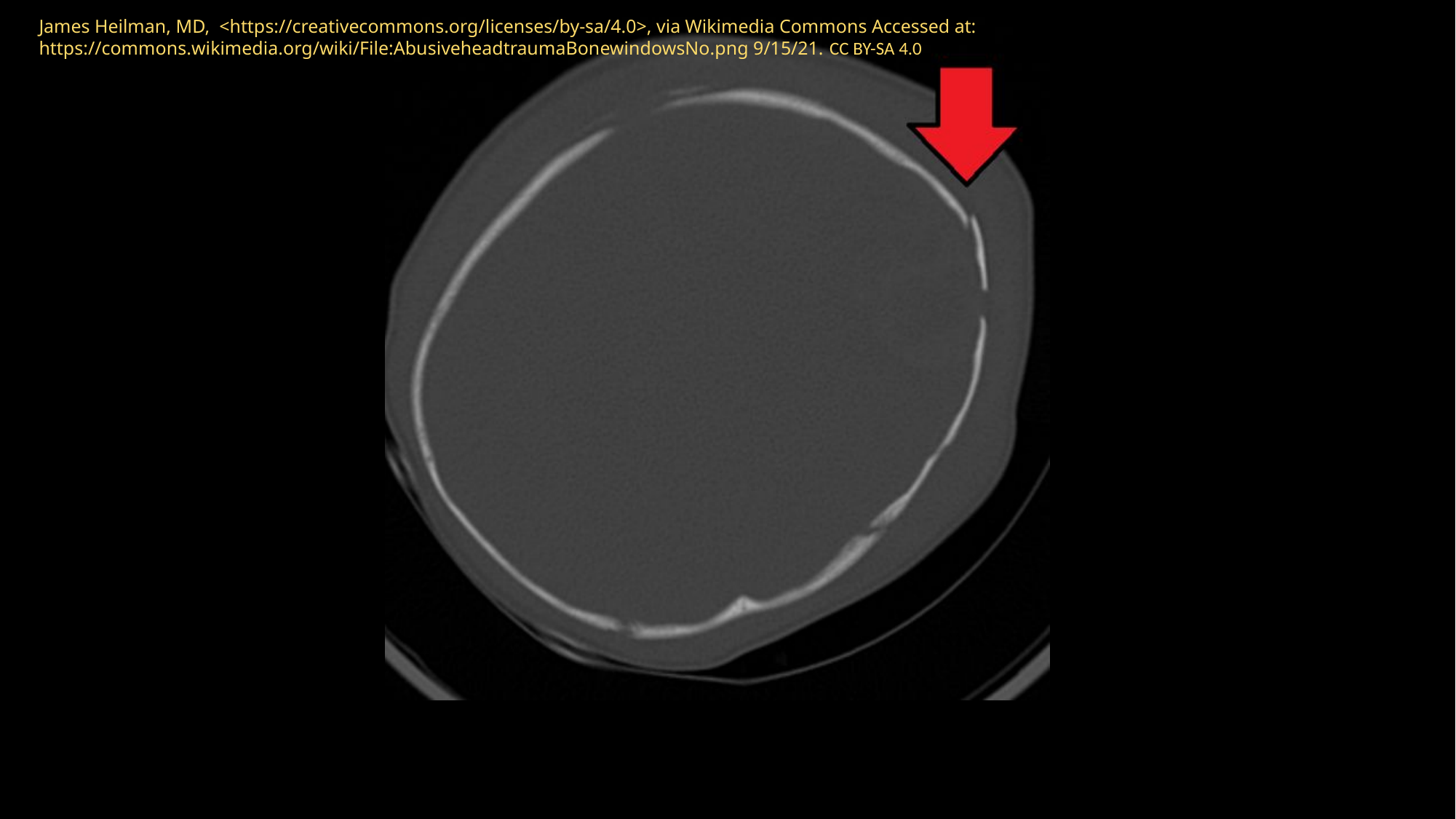

James Heilman, MD,  <https://creativecommons.org/licenses/by-sa/4.0>, via Wikimedia Commons Accessed at: https://commons.wikimedia.org/wiki/File:AbusiveheadtraumaBonewindowsNo.png 9/15/21. CC BY-SA 4.0

## Slide 49
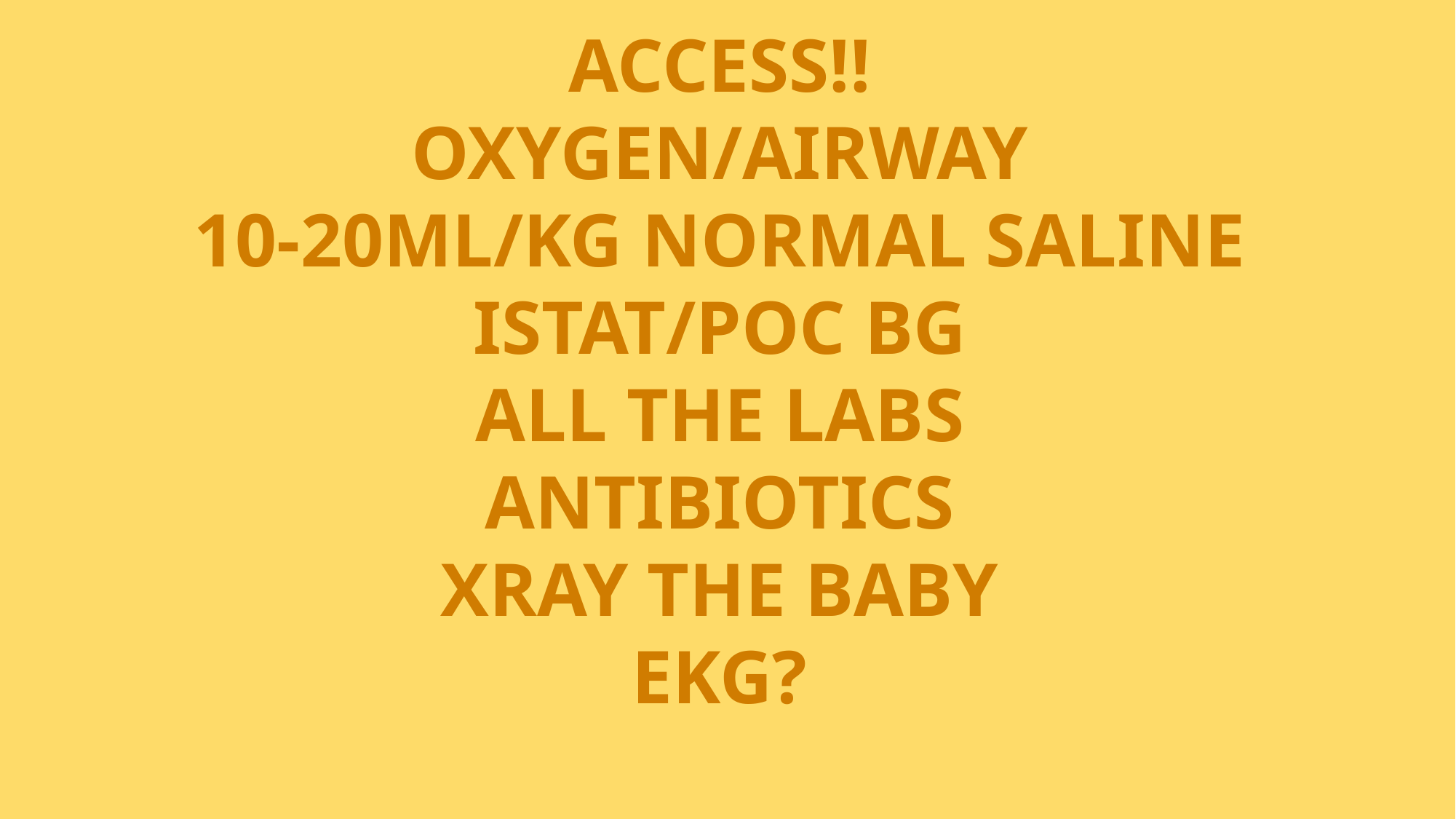

ACCESS!!
OXYGEN/AIRWAY
10-20ML/KG NORMAL SALINE
ISTAT/POC BG
ALL THE LABS
ANTIBIOTICS
XRAY THE BABY
EKG?

## Slide 50
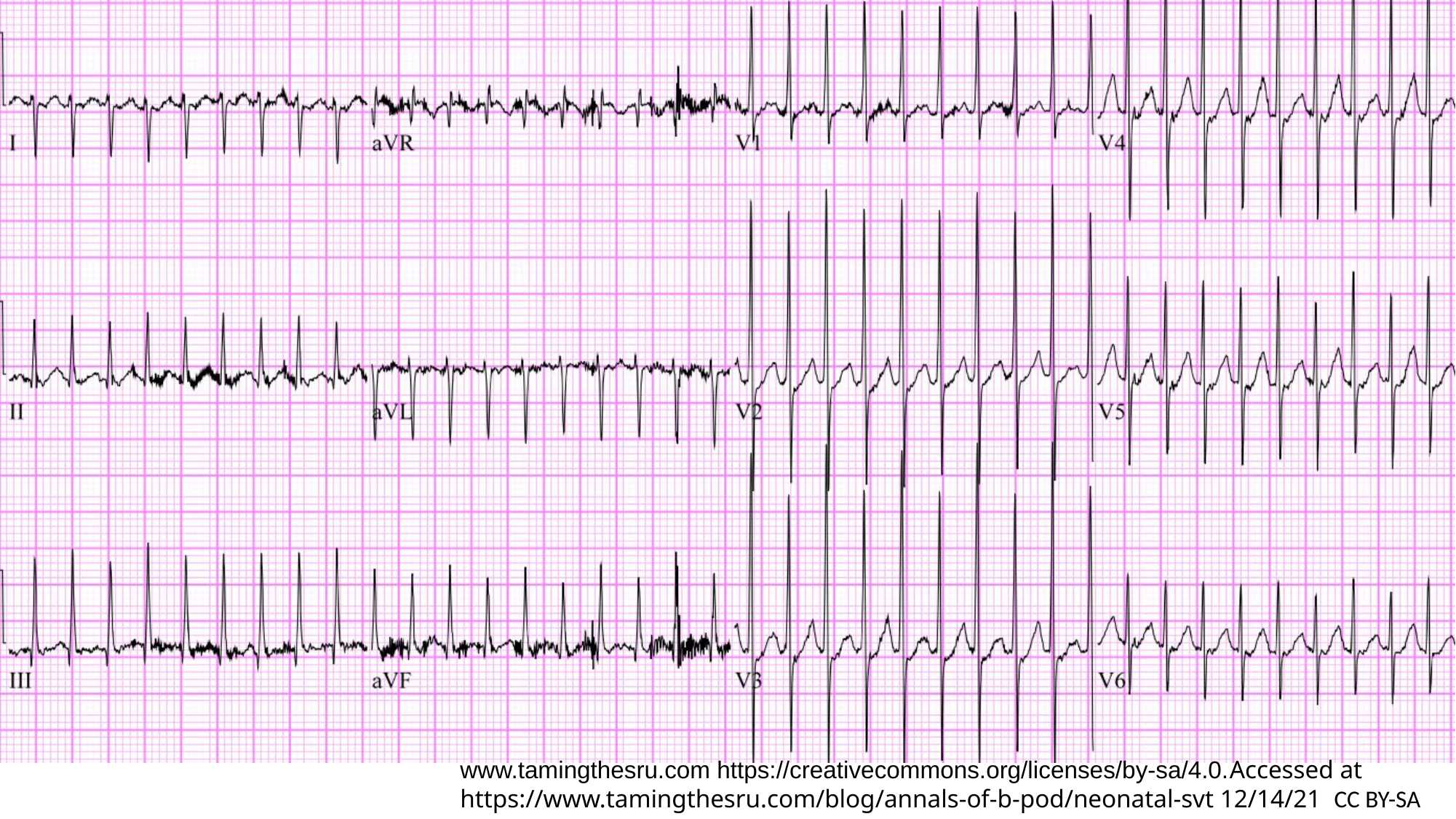

www.tamingthesru.com https://creativecommons.org/licenses/by-sa/4.0.Accessed at https://www.tamingthesru.com/blog/annals-of-b-pod/neonatal-svt 12/14/21  CC BY-SA 4.0

## Slide 51
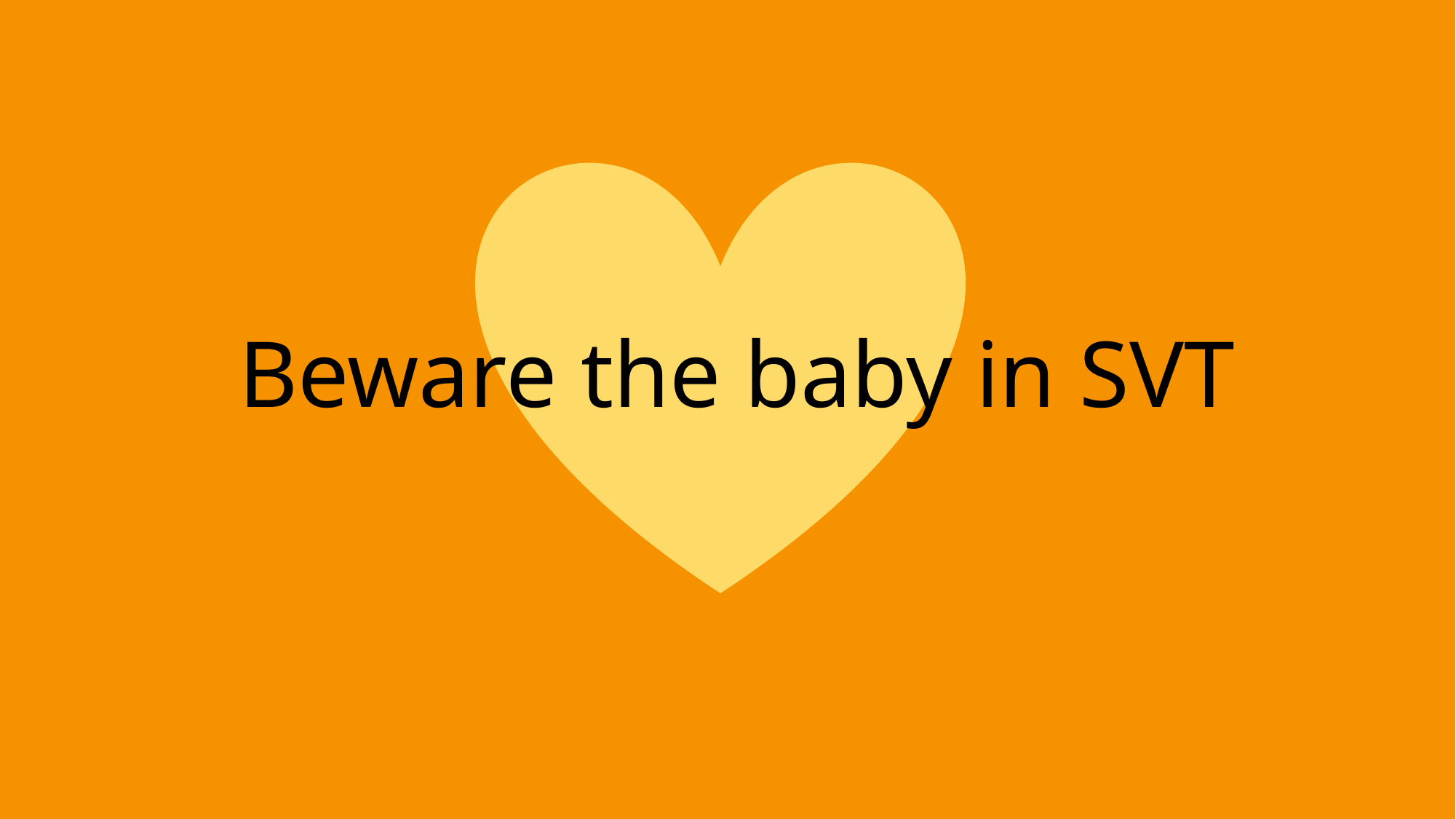

Beware the baby in SVT

## Slide 52
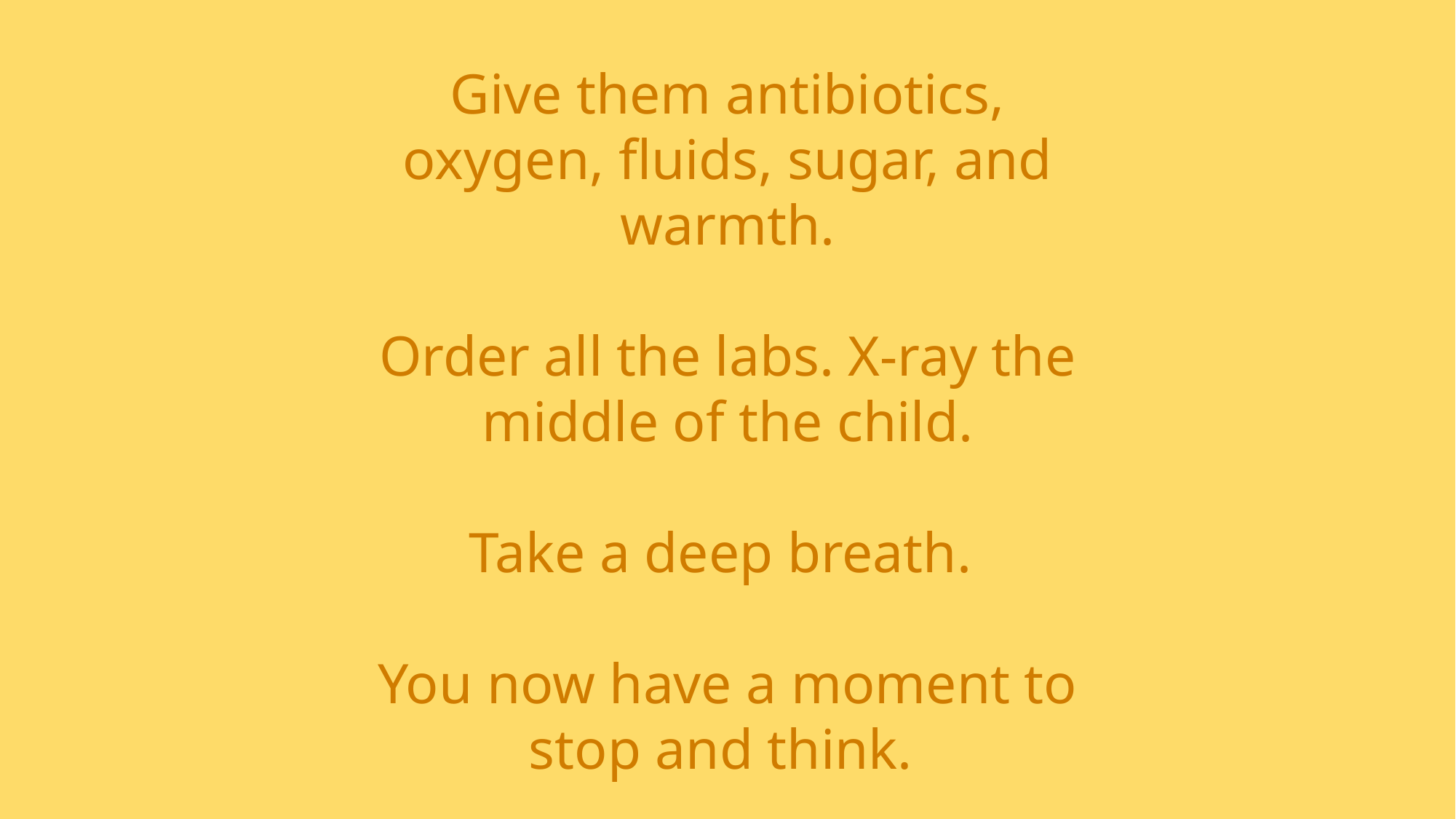

Give them antibiotics, oxygen, fluids, sugar, and warmth.
Order all the labs. X-ray the middle of the child.
Take a deep breath.
You now have a moment to stop and think.

## Slide 53
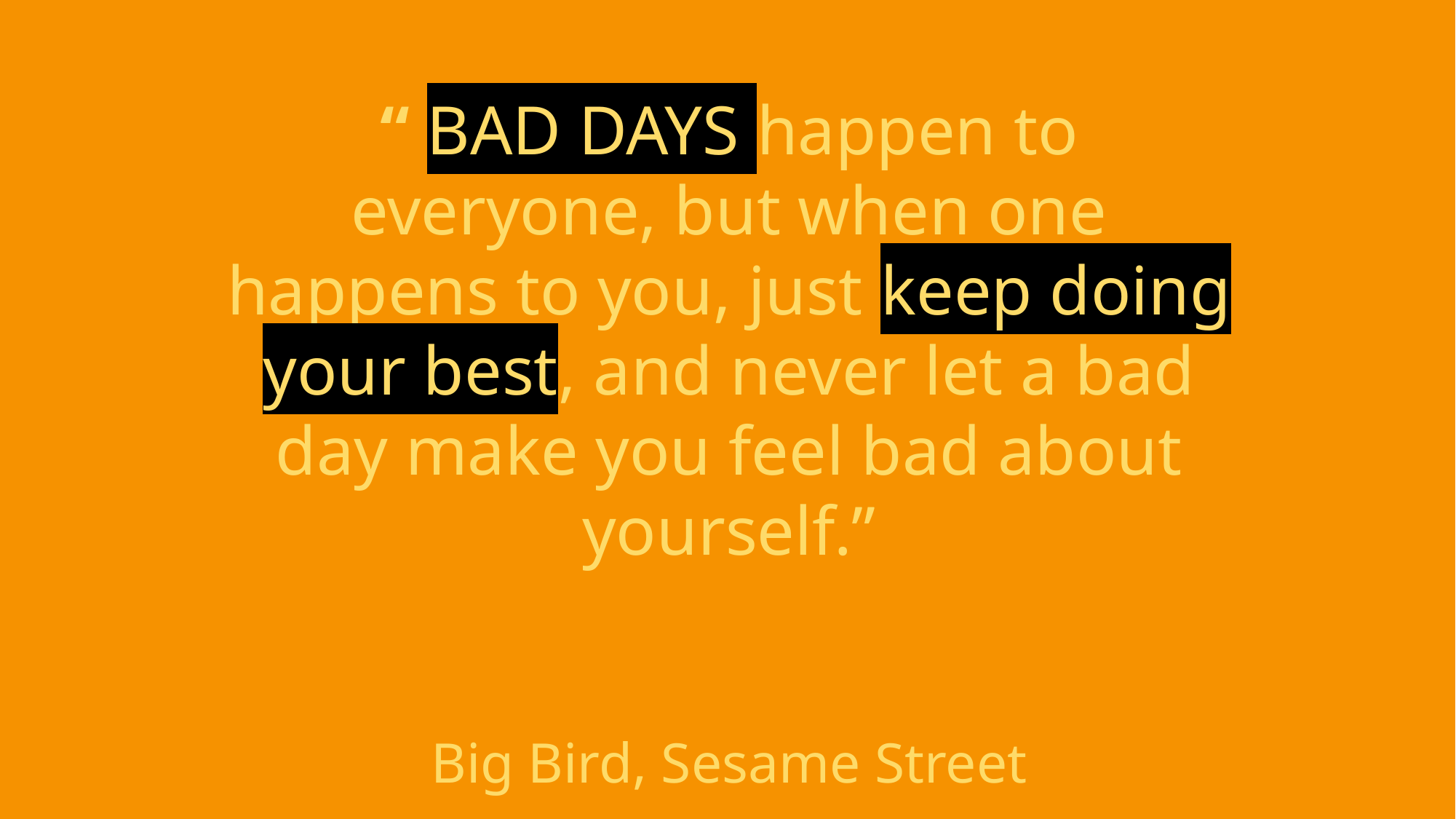

“ BAD DAYS happen to everyone, but when one happens to you, just keep doing your best, and never let a bad day make you feel bad about yourself.”
Big Bird, Sesame Street
